# Supplementary material for: Response thresholds alone cannot explain empirical patterns of division of labor in social insects
Source: PLoS Biol. 2021 Jun 17;19(6):e3001269. doi: 10.1371/journal.pbio.3001269 (PMC8211278; doi:10.1371/journal.pbio.3001269)
Supplement: S1 Text — (PDF) [file pbio.3001269.s001.pdf]

Supporting Information for

Response thresholds alone cannot explain empirical patterns of division of labor in social insects

Yuko Ulrich[a], Mari Kawakatsu[a], Christopher K. Tokita, Jonathan Saragosti, Vikram Chandra, Corina E. Tarnita[b,*], Daniel J. C. Kronauer[b,*]

[*] Corresponding authors: dkronauer@rockefeller.edu (DJCK), ctarnita@princeton.edu (CET)

Supplementary Analysis

1. Analytical treatment of the model

In addition to simulating the fixed threshold model described in Materials and Methods, we derive analytical predictions for its long-term behavior. We briefly summarize the key equations of the model as well as the simplifying assumptions we make in order to gain analytical insight.

Recall that the model considers a colony of N individuals performing M tasks; we assume that there are two tasks ($M = 2$; see model description). Individuals can be of one of two types, X or Y. To analytically study how individual behavior depends on the ratio of the types, we define f and $1 - f$ to be the fractions of the colony consisting of individuals of type X and Y, respectively.

The model assumes that individual i's internal threshold θ_{ij} is drawn from a normal distribution with mean μ_j and normalized standard deviation σ_j. For our analytical analysis, we assume that $\sigma_j = 0$ for all tasks. In other words, the type- and task-specific thresholds are assumed to be given by the constant parameters, μ_j^X and μ_j^Y. Under this assumption, the probabilities $P_{ij,t}^X$ and $P_{ij,t}^Y$ that inactive individuals i of types X and Y begin to perform task j at time t are, respectively,

$$P_{j,t}^X(s_{j,t}) = \frac{s_{j,t}^\eta}{s_{j,t}^\eta + \left(\mu_j^X\right)^\eta}, \qquad P_{j,t}^Y(s_{j,t}) = \frac{s_{j,t}^\eta}{s_{j,t}^\eta + \left(\mu_j^Y\right)^\eta}. \tag{1}$$

Because we assume that there are two tasks ($M = 2$), the numbers of X and Y individuals performing task j at time $t + 1$ are governed by the following equations, in which j' denotes the other task:

$$\begin{aligned} n_{j,t+1}^X - n_{j,t}^X &= \frac{1}{2}\left[P_{j,t}^X(s_{j,t}) + \left(1 - P_{j',t}^X(s_{j',t})\right) P_{j,t}^X(s_{j,t})\right]\left(fN - (n_{j,t}^X + n_{j',t}^X)\right) - \tau n_{j,t}^X \\ n_{j,t+1}^Y - n_{j,t}^Y &= \frac{1}{2}\left[P_{j,t}^Y(s_{j,t}) + (1 - P_{j',t}^Y(s_{j',t}))P_{j,t}^Y(s_{j,t})\right]\left((1-f)N - (n_{j,t}^Y + n_{j',t}^Y)\right) - \tau n_{j,t}^Y, \end{aligned} \tag{2}$$

where $n_{j,t}^X$ and $n_{j,t}^Y$ are the numbers of X and Y individuals performing task j at time t, respectively, and τ is the probability of quitting a task. The sums in the larger parentheses represent the pool of individuals who could possibly initiate task j—that is, the total number of inactive individuals. The sums in square brackets then capture the possible ways in which these inactive individuals can initiate task j: they can either encounter the stimulus for task j immediately and begin performing that task, or they can first encounter the stimulus for the other task j', not perform that task, subsequently encounter the stimulus for task j, and begin performing task j. Lastly, recall that the dynamics of stimulus $s_{j,t}$ associated with task j is governed by Eq. (3):

$$s_{j,t+1} - s_{j,t} = \delta_j - \frac{\alpha_j^X n_{j,t}^X + \alpha_j^Y n_{j,t}^Y}{N}, \tag{3}$$

where δ_j is the task-specific demand rate, and α_j^X and α_j^Y are the task-specific performance efficiencies of X and Y individuals, respectively.

In the subsequent sections, we compute the long-term behavior of the system of six difference equations in Eq. (2) (for $n_1^X, n_1^Y, n_2^X, n_2^Y$) and Eq. (3) (for s_1, s_2) and compare the results to simulations.

A. Steady-state predictions.

Theoretical maximum activity level. In the model, individuals have a latency period of one time step between when they quit a task and when they recommence working. This means that, on average, only a fraction of the colony can be working at any given time.

To find this maximum activity level, let $Z_t = (n^X_{1,t} + n^Y_{1,t} + n^X_{2,t} + n^Y_{2,t})/N$ be the fraction of active individuals in a colony at time t. Note that $0 \leq Z_t \leq 1$. At time $t+1$, on average, a fraction τZ_t of the colony becomes inactive. Therefore, at time $t+1$,

$$\text{(fraction active)} + \text{(fraction inactive)} = X_{t+1} + \tau Z_t \leq 1.$$

At steady state, the equality $X_{t+1} = X_t = Z^*$ is satisfied. By substitution, we obtain the theoretical maximum activity level:

$$Z^* \leq \frac{1}{1+\tau}. \tag{4}$$

For example, for $\tau = 0.2$ used in the simulations (see Fig 3), at most 83.33% of the individuals in a colony can be active at steady state. A similar condition has been noted by [S1].

Pure colonies. Without loss of generality, we consider pure colonies that consist of type X individuals only: let $f = 1$ and $n^Y_{j,t} = 0$ for all t. By setting Eqs. (2) and (3) to zero, we obtain the fraction of X individuals performing task j at steady state, given by

$$\frac{n^X_j}{N} = \frac{\delta_j}{\alpha^X_j}. \tag{5}$$

Notably, the steady-state values of n^X_j are independent of the mean threshold (μ^X_j) or the quit probability (τ^X). This agrees with our simulation results in which differences in μ (Fig 1c) or τ (S5 Fig) alone did not change the mean task performance levels in pure colonies.

According to the condition in (4), this steady state is biologically possible only if

$$(Z^* =)\ \frac{n^X_1}{N} + \frac{n^X_2}{N} = \frac{\delta_1}{\alpha^X_1} + \frac{\delta_2}{\alpha^X_2} \leq \frac{1}{1+\tau}.$$

If this condition is not met, then the stimuli are expected to continue growing (i.e., system would not reach a steady state).

Now, suppose that the demand rate and task performance efficiency are the same for both tasks ($\delta_1 = \delta_2 = \delta$, $\alpha^X_1 = \alpha^X_2 = \alpha^X$). Equation (5) implies that the fractions of X individuals performing tasks 1 and 2 at steady state would be

$$\frac{n^X_1}{N} = \frac{n^X_2}{N} = \frac{\delta}{\alpha^X}.$$

Similarly, in pure colonies of type Y, if $\delta_1 = \delta_2 = \delta$ and $\alpha^Y_1 = \alpha^Y_2 = \alpha^Y$, then $n^Y_1/N = n^Y_2/N = \delta/\alpha^Y$ at steady state. Thus, in order for pure colonies of type X and type Y to have different average task performance levels (i.e., $\delta/\alpha^X \neq \delta/\alpha^Y$) under the assumptions above—i.e., that the tasks are equally demanding and that a given type of individual is equally efficient at both tasks—the two types must differ in task performance efficiency ($\alpha^X \neq \alpha^Y$) (see main text).

Mixed colonies with 1:1 mixes. We now consider mixed colonies consisting of X and Y individuals in equal proportions ($f = 0.5$). We assume that the mean thresholds and the quit probabilities are identical for both tasks and ant types ($\mu^X_1 = \mu^X_2 = \mu^Y_1 = \mu^Y_2$ and $\tau^X = \tau^Y$)[1]. Setting Eqs. (2) and (3) equal to zero, we find that the steady-state numbers of individuals performing task j are given by

$$n^X_j = n^Y_j = N\left(\frac{\delta_j}{\alpha^X_j + \alpha^Y_j}\right).$$

This quantity can also be expressed as a fraction of each type of individuals:

$$\frac{n^X_j}{(N/2)} = \frac{n^Y_j}{(N/2)} = \frac{2\delta_j}{\alpha^X_j + \alpha^Y_j}. \tag{6}$$

[1]The parameters μ and τ do not explicitly appear in Eq. (6) when we assume that the mean thresholds are identical for all individuals and both tasks. However, based on Eq. (2), we expect the general form of steady state fractions of active individuals to be explicit functions of μ^X_j and μ^Y_j as well as τ^X and τ^Y. While the steady states can be computed numerically for the case when these parameters differ between types or tasks, the analytical expressions are too complicated to write down.

Applying condition (4), this steady-state is only biologically relevant when

$$(Z^* =) \sum_{j=1}^{2} \frac{n_j^X}{N} + \frac{n_j^Y}{N} = \sum_{j=1}^{2} \frac{2\delta_j}{\alpha_j^X + \alpha_j^Y} \leq \frac{1}{1+\tau}. \tag{7}$$

Again, if this condition is not met, then we would expect the stimuli to continue growing over time (i.e., the colony is unable to keep up with the demand) and for the individuals to be working at maximum capacity.

Mixed colonies with non-1:1 mixes. We now generalize to the case in which a fraction f of individuals ($0 \leq f \leq 1$) in a mixed colony are of type X. In the simplified case where $\mu_1^X = \mu_2^X = \mu_1^Y = \mu_2^Y$ and $\tau^X = \tau^Y$, the steady-state fractions of individuals performing task j are

$$n_j^X = \frac{fn\delta_j}{f\alpha_j^X + (1-f)\alpha_j^Y}, \quad n_j^Y = \frac{(1-f)n\delta_j}{f\alpha_j^X + (1-f)\alpha_j^Y}.$$

Since there are fn individuals of type X and $(1-f)n$ individuals of type Y, these quantities can be expressed as fractions of individuals of type X and Y individuals performing task j:

$$\frac{n_j^X}{fN} = \frac{n_j^Y}{(1-f)N} = \frac{\delta_j}{f\alpha_j^X + (1-f)\alpha_j^Y} \left(= \frac{n_j^X + n_j^Y}{N} \right). \tag{8}$$

The last equality highlights the fact that, at steady state, the fraction of individuals of each type performing task j is identical to the fraction of the whole colony performing that task, i.e., both X and Y perform task j at equal rates. As expected, the expressions Eq. (8) reduce to Eq. (6) when $f = 0.5$ (1:1 mixes) and to Eq. (5) when $f = 1$ (pure colonies with X individuals only). Again, we expect to see this equilibrium only when condition (cond. (4)) is satisfied. Moreover, from Eq. (8), we expect the steady-state task j performance frequency to depend non-linearly on the fraction f of X individuals.

Mixed colonies with symmetric mean thresholds. So far we have assumed that the mean task thresholds μ_j^X and μ_j^Y are identical for both ant types and tasks ($\mu_1^X = \mu_2^X = \mu_1^Y = \mu_2^Y$). While Eqs. (2) and (3) can be solved numerically when we introduce between-type differences in μ, the steady-state expressions become too difficult to write down. In the following special case, however, we can express the steady-state values exactly. Assume that

1. colonies consist of type X and Y individuals in equal proportions ($f = 0.5$);
2. task efficiency is the same for both ant types and tasks ($\alpha_1^X = \alpha_2^X = \alpha_1^Y = \alpha_2^Y = \alpha$);
3. demand rate is the same for both tasks ($\delta_1 = \delta_2 = \delta$); and
4. mean task thresholds are symmetric, such that one type has a low threshold for one task and a high threshold for the other while this ordering is reversed in the other type: $\mu_1^X = \mu_2^Y = a$ and $\mu_2^X = \mu_1^Y = b$.

Importantly, the symmetry between the two tasks and between the two types imply that the stimulus levels for the tasks would be identical at stead state ($s_1 = s_2 = s^*$). Moreover, at steady state, the number of X individuals performing task 1 would be identical to the number of Y individuals performing task 2 ($n_1^X = n_2^Y$); similarly, we would expect that $n_1^Y = n_2^X$. Substituting these conditions into Eq. (3) and setting it equal to zero, we find that, at steady state,

$$n_1^X + n_1^Y = n_2^X + n_2^Y = n_1^X + n_2^X = n_1^Y + n_2^Y = N\left(\frac{\delta}{\alpha}\right).$$

By substituting this into Eq. (2) and following the symmetry argument above, we derive an expression for the steady-state stimulus level s^*:

$$s^*(= s_1 = s_2) = \left[\frac{1}{2}\left(-(a^\eta + b^\eta) \pm \sqrt{(a^\eta + b^\eta)^2 + (a^\eta b^\eta) \cdot \frac{8\delta\tau}{\alpha - 2\delta(1+\tau)}}\right)\right]^{\frac{1}{\eta}}.$$

The corresponding steady-state fractions of X and Y individuals performing tasks 1 and 2 are

$$\begin{aligned} \frac{n_1^X}{(N/2)} &= \frac{n_2^Y}{(N/2)} = \frac{1}{\tau}\left(\frac{(s^*)^\eta}{(s^*)^\eta + a^\eta}\right)\left[2 - \frac{(s^*)^\eta}{(s^*)^\eta + b^\eta}\right]\left(\frac{1}{2} - \frac{\delta}{\alpha}\right), \\ \frac{n_2^X}{(N/2)} &= \frac{n_1^Y}{(N/2)} = \frac{1}{\tau}\left(\frac{(s^*)^\eta}{(s^*)^\eta + b^\eta}\right)\left[2 - \frac{(s^*)^\eta}{(s^*)^\eta + a^\eta}\right]\left(\frac{1}{2} - \frac{\delta}{\alpha}\right). \end{aligned} \tag{9}$$

When $a = b$ (i.e., when all μ's are identical), these expressions reduce to the steady-states predicted in Eq. (6).

B. Downward vs. upward convergence. Both our experiments (Fig 2a) and theoretical analyses (Fig 3a-b) demonstrated patterns of asymmetric behavioral convergence between the types, in which individuals of different types were behaviorally more similar to each other when mixed. Here we combine our analytical predictions for pure and mixed colonies to investigate conditions under which such convergence patterns arise. Consider two pure colonies consisting of X and Y individuals, respectively, and a third, mixed colony consisting of a 1:1-ratio of X and Y individuals. Let us assume that each colony reaches a steady state (i.e., each colony satisfies cond. (4)). We show analytically that, under these conditions, if the ant types only differ in task efficiency (α_j^X, α_j^Y), then the system can exhibit a downward convergence but not an upward convergence.

We can directly apply the steady-state fractions of active individuals in Eqs. (5) and (6) because the mean threshold (μ) and the quit probability (τ) are assumed to be identical across types. The behavioral convergence is *downward* if

$$\frac{1}{2}\left(\frac{\delta_j}{\alpha_j^X}+\frac{\delta_j}{\alpha_j^Y}\right) > \frac{2\delta_j}{\alpha_j^X+\alpha_j^Y} \tag{10}$$

and *upward* if the inequality is reversed (see also Fig 3a-b).

By manipulating the inequality (10), we see that the left-hand side is always at least as large as the right-hand side:

$$\frac{1}{2}\left(\frac{\delta_j}{\alpha_j^X}+\frac{\delta_j}{\alpha_j^Y}\right) - \frac{2\delta_j}{\alpha_j^X+\alpha_j^Y} = \frac{\delta_j}{2}\left(\frac{(\alpha_j^X-\alpha_j^Y)^2}{\alpha_j^X\alpha_j^Y(\alpha_j^X+\alpha_j^Y)}\right) \geq 0$$

The equality holds if and only if $\alpha_j^X = \alpha_j^Y$, in which case the types are indistinguishable with respect to task j. If $\alpha_j^X \neq \alpha_j^Y$, then only downward convergence is possible under our assumptions (in particular, we assume that condition Eq. (4) is satisfied). Note that the threshold between upward and downward convergence, Eq. (10) is agnostic to between-task differences in task efficiency or task demand; in other words, it holds even when $\alpha_1^X \neq \alpha_2^X$, $\alpha_1^Y \neq \alpha_2^Y$, and $\delta_1 \neq \delta_2$.

C. Contextualizing the analytical calculations. To put our analytical results into the context of the main text, consider scenarios (a) with two high-demand tasks (e.g., $\delta_1 = \delta_2 = 1.3$, as in Fig 3a), (b) with two low-demand tasks (e.g., $\delta_1 = \delta_2 = 0.6$, as in Fig 3b), and (c) with one high-demand task and one low-demand task (e.g., $\delta_1 = 1.3$, $\delta_2 = 0.6$). We suppose that all type-specific parameters are identical across scenarios and that mean thresholds and quit probabilities are identical for both tasks and ant types (i.e., $\mu_1^X = \mu_2^X = \mu_1^Y = \mu_2^Y, \tau^X = \tau^Y$, as in *Mixed colonies with 1:1 mixes*).

Equation (6) and condition (7) (or, equivalently, cond. (4)) suggest that, in the absence of differences in mean threshold, the total task performance frequency of all ants in a colony (i.e., $\sum_{j=1}^{2} 2\delta_j/(\alpha_j^X+\alpha_j^Y)$) would be highest in (a), lowest in (b), and intermediate in (c); and that a colony is most likely to keep up with the demand in (c), less likely in (b), and least likely in (a). In this sense, we predict that the outcome in (c) will be quantitatively intermediate between (a) and (b). However, this would not alter the possible qualitative outcomes of mixing: if condition (7) is satisfied under scenario (c), we would expect mixing to produce a downward convergence (see *Downward vs. upward convergence*); if not, mixing could lead to either downward or upward convergence depending on demand values. See also *An expanded model of DOL* in the main text.

2. Theoretical predictions for mean task performance in non-1:1 mixes

We further explored expected patterns of task allocation in colonies with different ratios of ant types. For the parameter combinations in Fig 3—which collectively captured all experimentally observed patterns—we investigated how the mean task performance of colonies changed as we varied the ratio of the two ant types.

Simulations predicted a striking range of patterns. For the parameter combination that produced no effect in the mixed colonies with equal proportions of the two ant types ('1:1 mixes'), the model produced an approximately linear relationship between mean task performance and the ratio of ant types (S7a Fig). In all other cases, the mean task performance followed nonlinear functions the ratio of the types, but their shapes differed among the cases. In the cases corresponding to behavioral convergence in the 1:1 mixes, the relationship followed a convex decreasing function, so long as there were enough individuals of the more efficient type such that the colony could keep up with the demand (S7b Fig; Supplementary Analysis 1); otherwise the colony performed the tasks at a fixed maximum capacity that depended only on the average task duration (S7c Fig). In the case corresponding to behavioral divergence, the relationship followed a concave decreasing function (S7d Fig). Hence, despite one type being more efficient than the other in all cases considered, replacing an individual of the former type with one of the latter type would lead to qualitatively different outcomes depending on the differences in mean threshold.

Regardless of the case studied, the ratio of the types did not alter the qualitative effect of mixing on individual behavior (behavioral convergence, divergence, or no effect); for example, the case that led to behavioral divergence in 1:1 mixes predicted behavioral divergence for all non-1:1 mixes tested (S7d Fig).

Reference

1. Gautrais J, Theraulaz G, Deneubourg JL, Anderson C. Emergent polyethism as a consequence of increased colony size in insect societies. J Theor Biol. 2002;215(3):363–373.
