# Supplementary figures and images for: Response thresholds alone cannot explain empirical patterns of division of labor in social insects
Source: PLoS Biol. 2021 Jun 17;19(6):e3001269. doi: 10.1371/journal.pbio.3001269 (PMC8211278; doi:10.1371/journal.pbio.3001269)

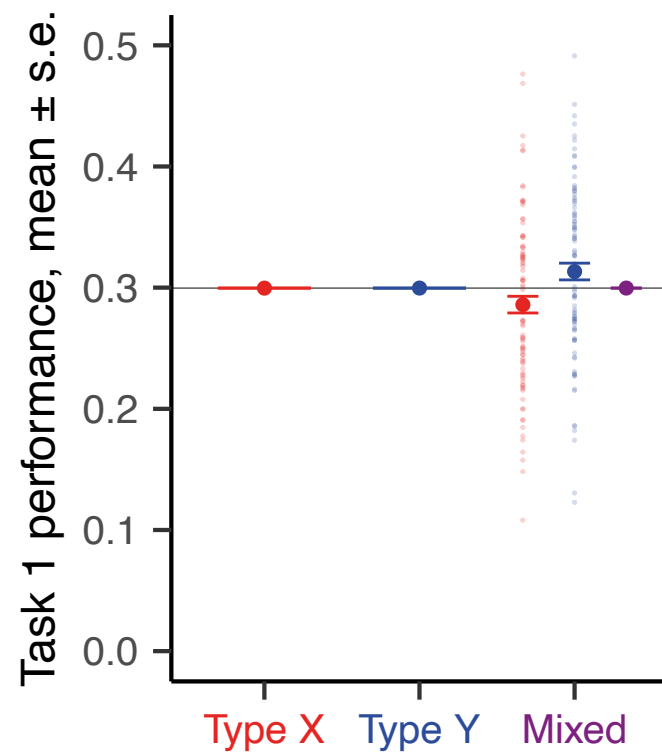

Supplement: S1 Fig — Task performance frequency for a single task as a function of colony composition. Opaque circles represent individual replicate colonies (N = 16; n = 100 replicates per composition), and solid circles represent average value (± s.e.) across replicates. Horizontal gray lines represent the average of the pure colonies (first two columns). Types X and Y differ in threshold variance: σX = 0.1, σY = 0.5; all other parameters are identical (see S1 Table). Simulation code and data are available at https://github.com/marikawakatsu/mixing-model. (PDF) [file pbio.3001269.s002.pdf]

**a**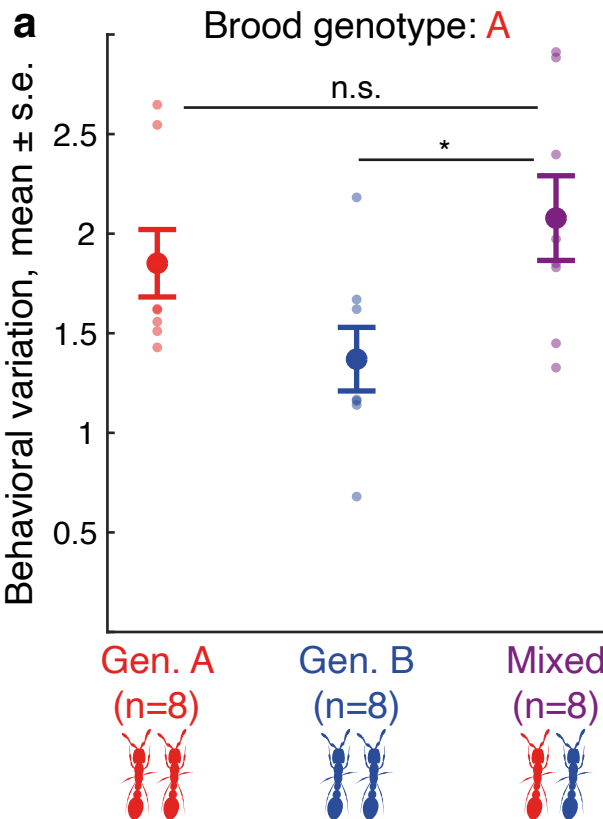**b**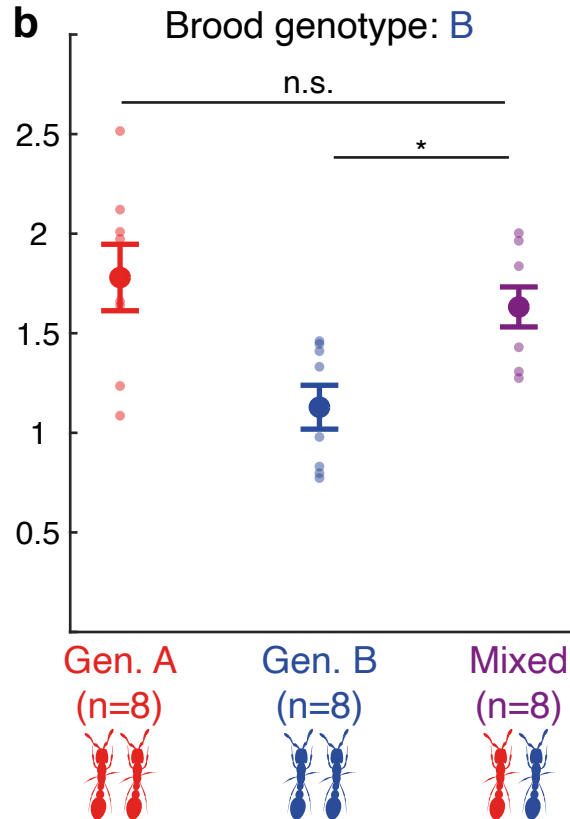**c**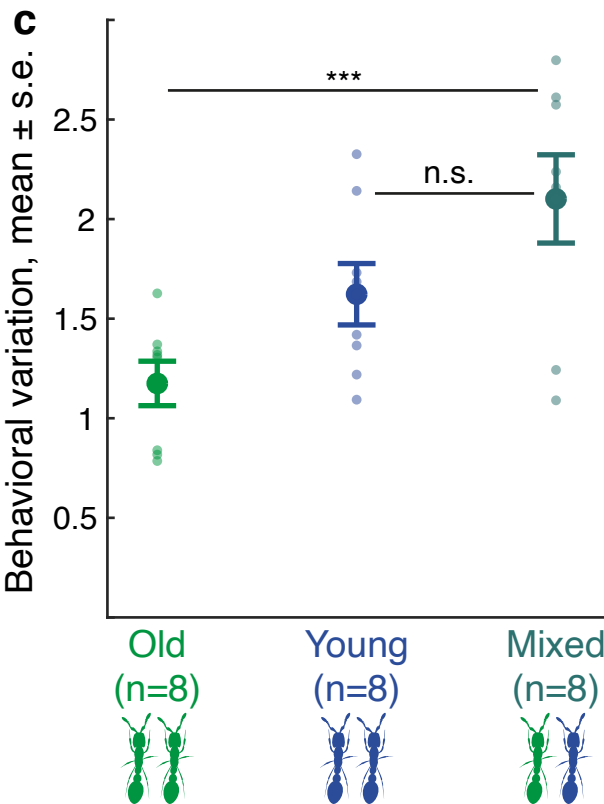**d**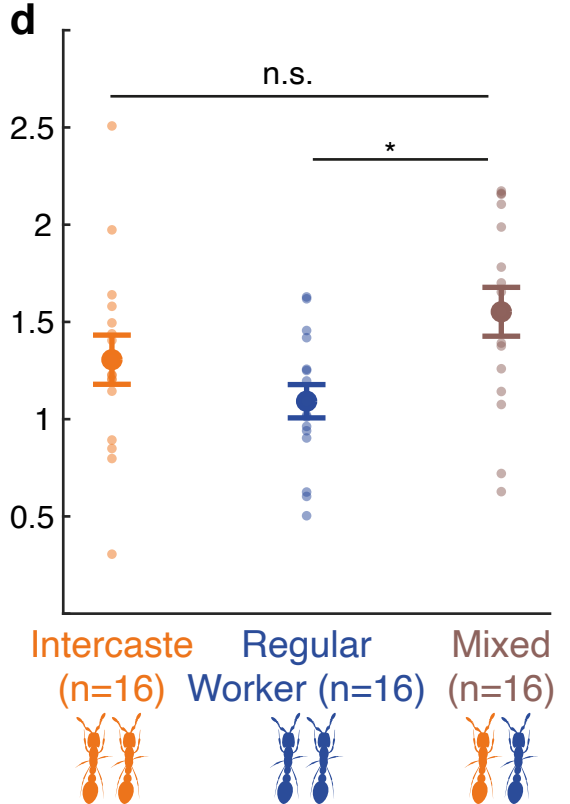

Supplement: S2 Fig — Small opaque circles represent individual colonies, and large solid circles represent the average values across n replicate colonies. Identical colors across panels indicate ants of the same genotype, age, and morphological types. (a) Behavioral variation as a function of colony genetic composition in colonies with A brood (N = 16; Bpure vs. Mixed: z = −2.85, p = 0.013; Apure vs. Mixed: z = 0.81, p = 0.421). (b) Behavioral variation as a function of colony genetic composition in colonies with B brood (N = 16; Bpure vs. Mixed: z = −2.76, p = 0.012; Apure vs. Mixed: z = −0.81, p = 0.419). (c) Behavioral variation as a function of colony age composition (N = 16; Youngpure vs. Mixed: z = 2.01, p = 0.090; Oldpure vs. Mixed: z = 3.89, p = 3.07*10−04). (d) Behavioral variation as a function of colony morphological composition (N = 8; Regular Workerpure vs. Mixed: z = −2.85, p = 0.013, Intercastepure vs. Mixed: z = 1.53, p = 0.254). n.s., nonsignificant; *, p < 0.05; **, p < 0.01; ***, p < 0.001. Raw data are available at doi.org/10.5061/dryad.hx3ffbgdd. r.m.s.d., root–mean–square deviation. (PDF) [file pbio.3001269.s003.pdf]

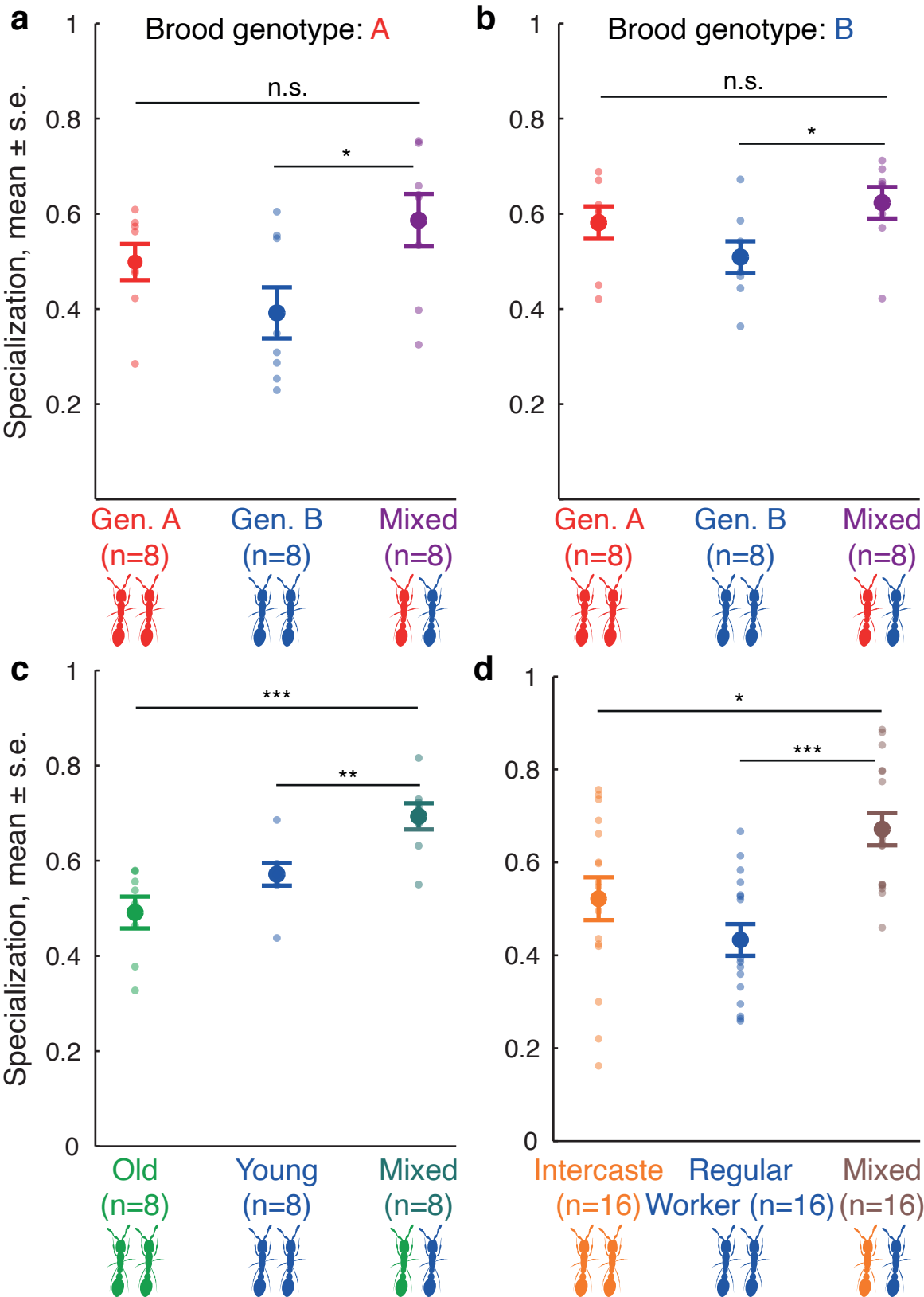

Supplement: S3 Fig — Small opaque circles represent individual colonies, and large solid circles represent the average values across n replicate colonies. Identical colors across panels indicate ants of the same genotype, age, and morphological types. (a) Specialization as a function of colony genetic composition in colonies with A brood (N = 16; GLM post hoc tests; Bpure vs. Mixed: z = −2.78, p = 0.017; Apure vs. Mixed: z = 1.25, p = 0.256). (b) Specialization as a function of colony genetic composition in colonies with B brood (N = 16; Bpure vs. Mixed: z = −2.41, p = 0.048; Apure vs. Mixed: z = 0.88, p = 0.378). (c) Specialization as a function of colony age composition (N = 16; Youngpure vs. Mixed: z = 3.01, p = 0.005; Oldpure vs. Mixed: z = 5.01, p = 1.63*10−06). (d) Specialization as a function of colony morphological composition (N = 8; Regular Workerpure vs. Mixed: z = −4.35, p = 4.07*10−05, Intercastepure vs. Mixed: z = 2.73, p = 0.013). n.s., nonsignificant; *, p < 0.05; **, p < 0.01; ***, p < 0.001. Raw data are available at doi.org/10.5061/dryad.hx3ffbgdd. r.m.s.d., root–mean–square deviation. (PDF) [file pbio.3001269.s004.pdf]

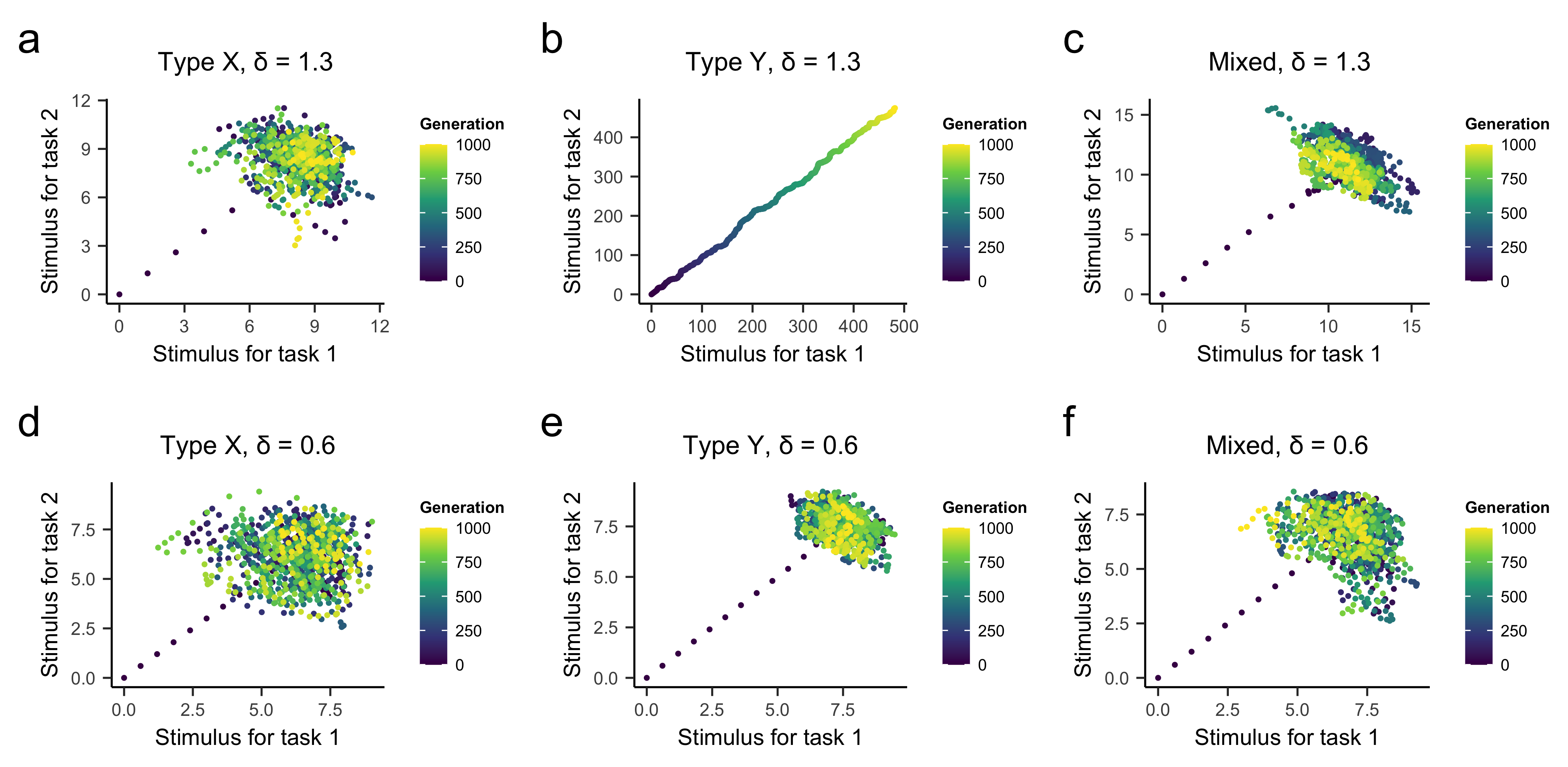

Supplement: S4 Fig — Each point shows the simulated stimulus level for the 2 tasks (task 1 on the horizontal axes and task 2 on the vertical axes) in the generation indicated by its color. Each of panels a, b, d, and e shows a pure colony of the type indicated; each of panels c and f shows a mixed colony of Types X and Y. Panels a–c (δ = 1.3) correspond to Fig 3A and d–f (δ = 0.6) to Fig 3B. (a–c)When the demand is higher (δ = 1.3), the more efficient type (Type X) can keep up with the demand on its own (a) but the less efficient type (Type Y) cannot, as demonstrated by the continual growth of the stimuli (b); however, mixed colonies can keep up with the higher level of demand (c). (d–f) When the demand is lower (δ = 0.6), the stimulus levels grow quickly at first but then stabilizes to an oscillatory pattern around a point, demonstrating that both pure and mixed colonies can keep up with the demand. Each simulation ran for 1,000 time steps; all other parameters are identical to those in the corresponding panels in Fig 3. Simulation code and data are available at https://github.com/marikawakatsu/mixing-model. (PNG) [file pbio.3001269.s005.png]

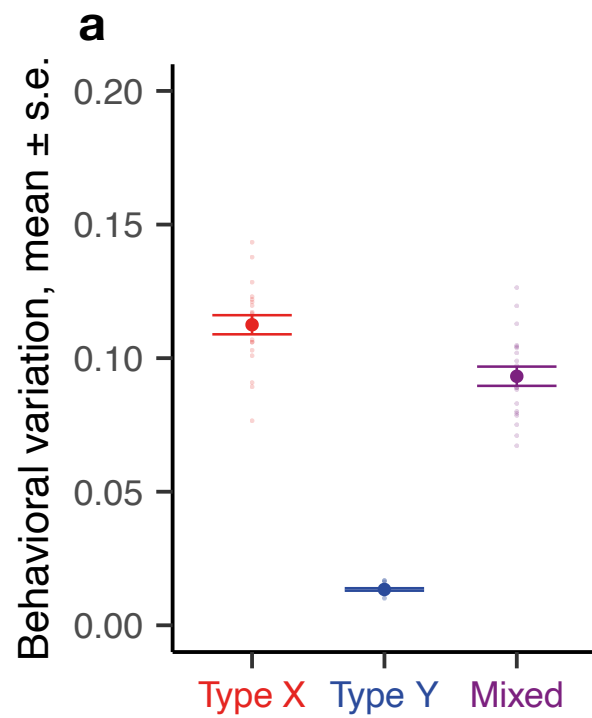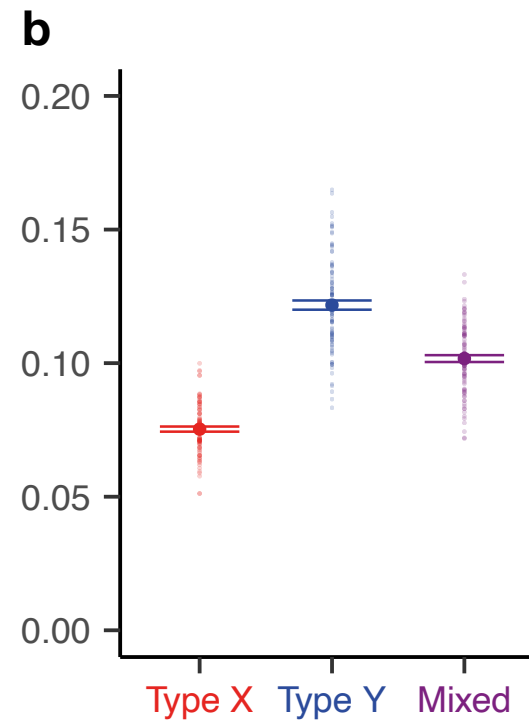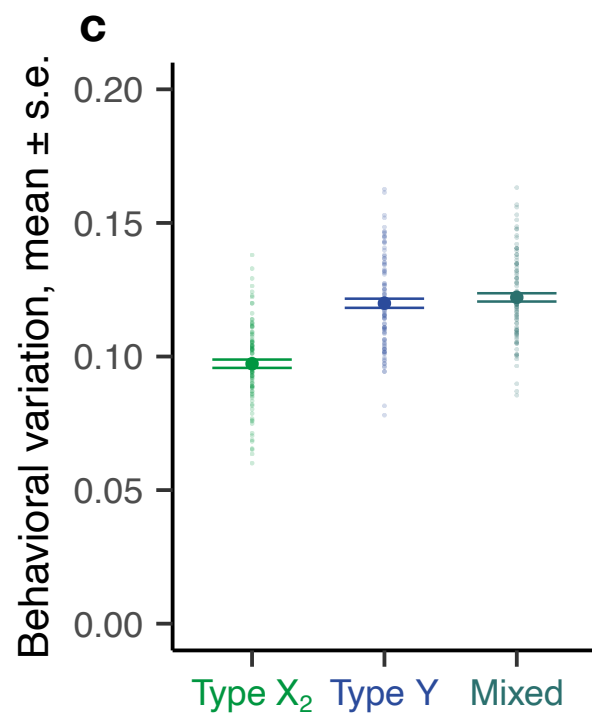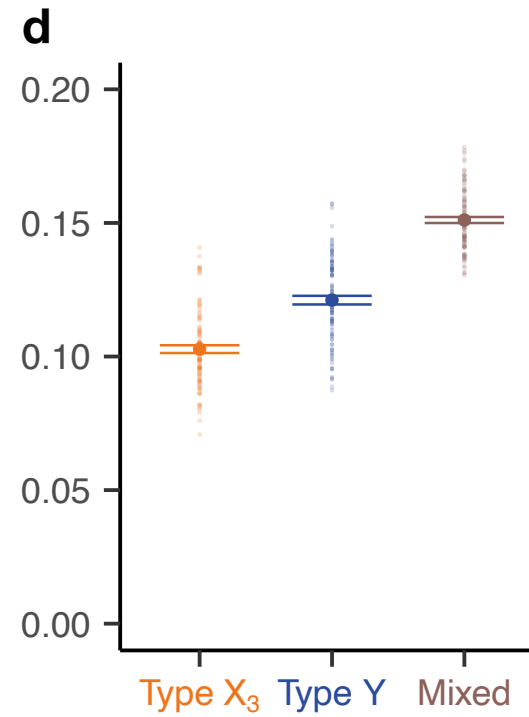

Supplement: S5 Fig — Behavioral variation was quantified as the standard deviation of task performance frequency across individuals in a colony. Opaque circles represent individual replicate colonies (N = 16; n = 100 replicates per composition), and solid circles represent the average value (mean ± s.e.) across replicates. Types X1, X2, X3, and Y and their corresponding parameters are identical to those in Fig 3. See S1 Table for other parameters. Simulation code and data are available at https://github.com/marikawakatsu/mixing-model. (PDF) [file pbio.3001269.s006.pdf]

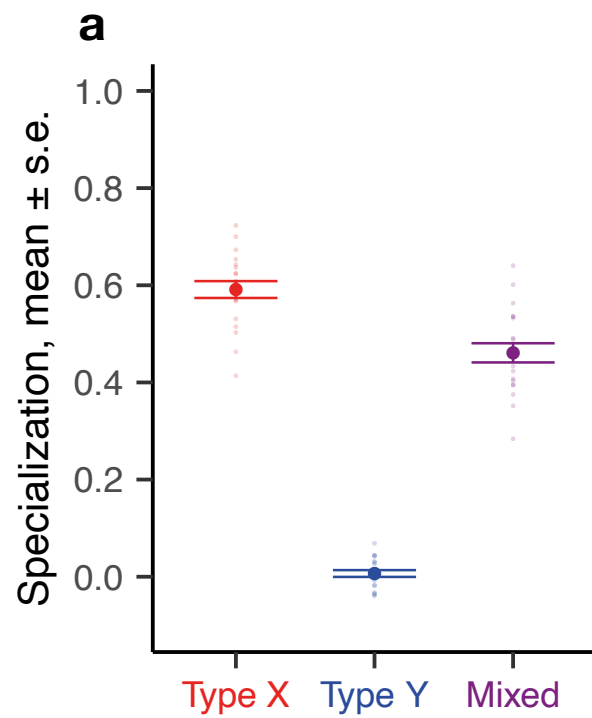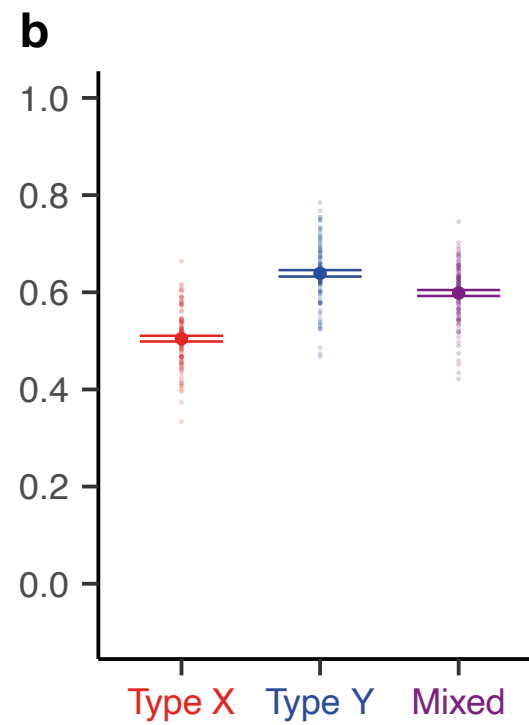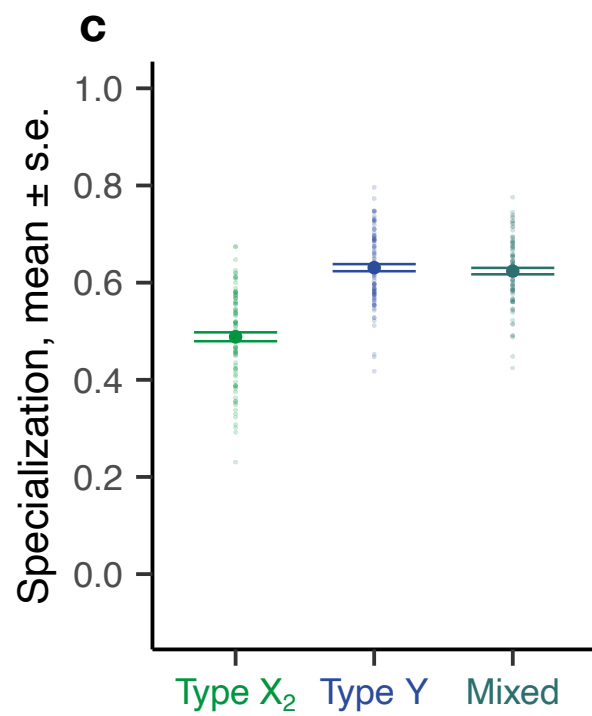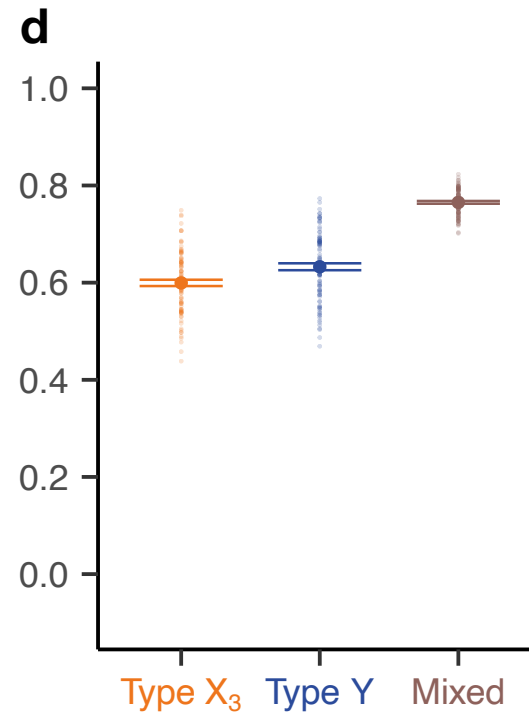

Supplement: S6 Fig — Colony-level specialization was quantified using Spearman rank correlation on consecutive windows of 200 time steps. Opaque circles represent individual replicate colonies (N = 16; n = 100 replicates per composition), and solid circles represent the average value (mean ± s.e.) across replicates. Types X1, X2, X3, and Y and their corresponding parameters are identical to those in Fig 3. See S1 Table for other parameters. Simulation code and data are available at https://github.com/marikawakatsu/mixing-model. (PDF) [file pbio.3001269.s007.pdf]

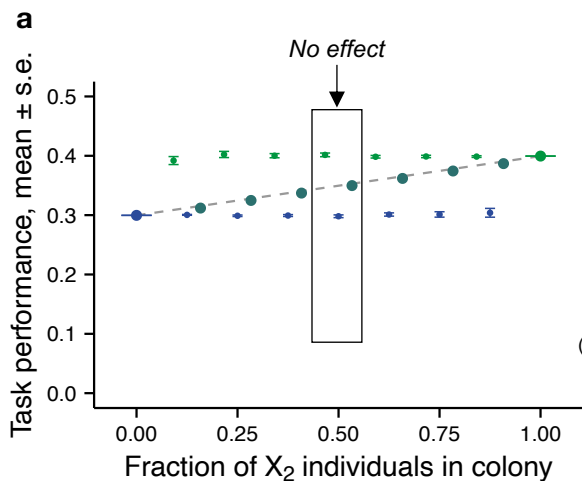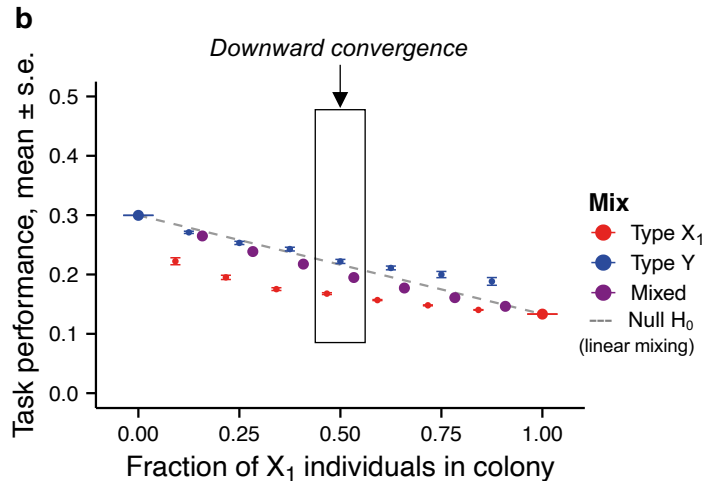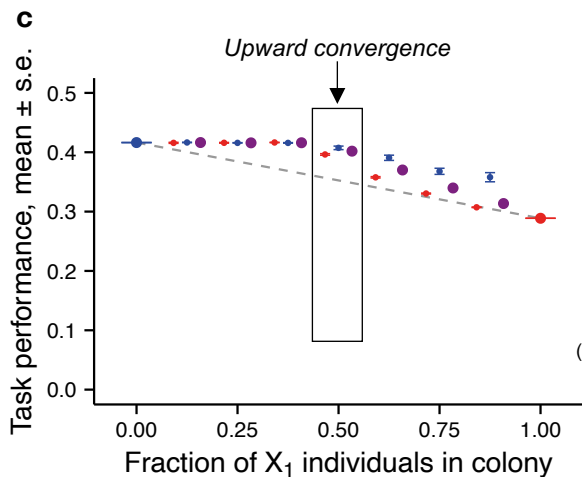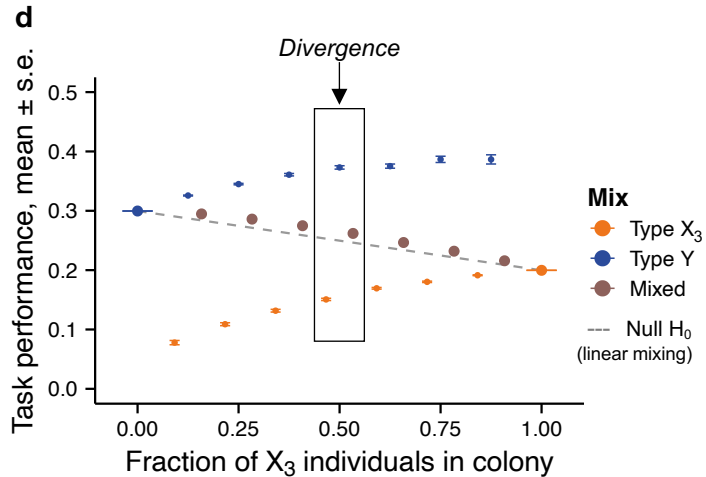

Supplement: S7 Fig — Colonies with varying ratios of X and Y individuals were simulated under different conditions of threshold values, task performance efficiency, and task demand (n = 100 replicates per colony composition). Each large circle represents the mean task performance (task 1) for that mix of X and Y individuals; the neighboring smaller circles represent the means of X and Y individuals, respectively, within that mix. Dashed lines indicate the null hypothesis of linear behavioral effects of mixing types. The boxes highlight the behavioral patterns characterizing the 1:1-mixes, and their labels indicate correspondence with Fig 3 (a: Fig 3E; b: Fig 3B; c: Fig 3A; and d: Fig 3D). Types X1, X2, X3, and Y and their corresponding parameters as in Fig 3. See S1 Table for other parameters. Simulation code and data are available at https://github.com/marikawakatsu/mixing-model. (PDF) [file pbio.3001269.s008.pdf]
