# Supplementary material for: Response thresholds alone cannot explain empirical patterns of division of labor in social insects
Source: PLoS Biol. 2021 Jun 17;19(6):e3001269. doi: 10.1371/journal.pbio.3001269 (PMC8211278; doi:10.1371/journal.pbio.3001269)
Supplement: S1 Table — (PDF) [file pbio.3001269.s009.pdf]

S1 Table. Parameter settings for model simulations.

Parameter	*Description*	*Baseline values*
T	Simulation length in time steps	10,000
N	Number of individuals	16
M	Number of tasks	2
$\delta_j = \delta$	Brood-specific rate of stimulus increase (i.e., demand rate); taken to be the same for all tasks	0.6
$\alpha_j^X = \alpha^X$<br>$\alpha_j^Y = \alpha^Y$	Type-specific performance efficiency of active individuals for task j; taken to be the same for all tasks	2
$\mu_j^X = \mu^X$<br>$\mu_j^Y = \mu^Y$	Mean of the type-specific threshold distribution for task j; taken to be the same for all tasks	10
$\sigma_j^X = \sigma^X$<br>$\sigma_j^Y = \sigma^Y$	Variance of the type-specific threshold distribution for task j as a fraction of the corresponding mean; taken to be the same for all tasks	0.1
η	Threshold stochasticity	7
$\tau_j^X = \tau^X$<br>$\tau_j^Y = \tau^Y$	Type-specific probability of quitting task j once active (inverse of average task performance duration); taken to be the same for all tasks	0.2
