# Supplementary material for: Response thresholds alone cannot explain empirical patterns of division of labor in social insects
Source: PLoS Biol. 2021 Jun 17;19(6):e3001269. doi: 10.1371/journal.pbio.3001269 (PMC8211278; doi:10.1371/journal.pbio.3001269)
Supplement: S2 Table — Text in bold denotes the variable of interest for each experiment. All mixed colonies contained a 1:1 ratio of each ant type. (PDF) [file pbio.3001269.s010.pdf]

S2 Table. List of experimental treatments. Text in bold denotes the variable of interest for each experiment. All mixed colonies contained a 1:1 ratio of each ant type.

Experiment	Worker genotype	Brood genotype	Age (cycles)	Subcaste	Colony size N	n replicates / composition	# colonies
Genetic composition 1	**A, B, mixed**	A	1	Regular workers	16	8	24
Genetic composition 2	**A, B, mixed**	B	1	Regular workers	16	8	24
Age composition	B	B	**1, 3, mixed**	Regular workers	16	8	24
Morphological composition	B	B	1	**Regular workers, intercastes, mixed**	8	16	48
