# Supplementary material for: Response thresholds alone cannot explain empirical patterns of division of labor in social insects
Source: PLoS Biol. 2021 Jun 17;19(6):e3001269. doi: 10.1371/journal.pbio.3001269 (PMC8211278; doi:10.1371/journal.pbio.3001269)
Supplement: Attachment — Submitted filename: Ulrich_Kawakatsu_etal_PLOSBio_ResponseLetter.pdf [file pbio.3001269.s011.pdf]

Dear Editors,

Thank you for the opportunity to revise and resubmit our manuscript PBIOLOGY-D-20-00979R1 to *PLOS Biology*. We are grateful for the Reviewers' constructive feedback and have carefully considered each of their comments. We believe that, as a result, our manuscript is greatly improved. Here, we provide a brief summary of how we have addressed each of the primary concerns of the Reviewers and of the Academic Editor, followed by our detailed responses below.

Reviewers 1 and 4 raised questions on the natural history of the clonal raider ant and on whether the size and composition of our experimental colonies were appropriate. We agree that additional details on the biology of our study system are important for background and context, and we have included additional information in the introduction. This also allowed us to explain in more detail why the experimental conditions used here are justified.

Comments from Reviewers 1 and 4 helped us realize that the theoretical model needed to be explained in more details in the main text. We have included a more detailed description of the model in the Results section, including key equations (as requested by Reviewer 4) and an explanation of the treatment of age in the model (as requested by Reviewer 1).

Reviewers 2 and 4 raised important points regarding the design, analysis, and interpretation of the experiments. We have added additional explanations and justifications of our experimental design and statistical approaches in Materials and Methods and moved most statistical results from figure legends to the main text, which we believe has improved the overall clarity of the results.

Reviewer 3 and the Academic Editor commented on the contextualization of the study. Some of the suggested references were in fact already cited in the original submission, but we have incorporated additional relevant references as suggested. The Academic Editor also pointed us to early work that we were not aware of, but greatly enjoyed reading and have now incorporated into the manuscript. This has helped us highlight how the current study builds on previous work and goes substantially beyond the state of the art.

Comments from Reviewers 3 and 4 as well as from the Academic Editor prompted us to expand the discussion to include several additional points. To address a comment from Reviewer 3 on the agreement between the experiments and the model, we now more comprehensively discuss our theoretical results and how they relate to the experiments. Following the Academic Editor's recommendation that the role of experience in modulating DOL—which we do not consider in the model—be addressed in the manuscript, we now explicitly discuss experience in the Conclusions. We hope that this more substantial discussion addresses the Reviewers concerns.

All Reviewer comments are reprinted below in plain text, followed by our responses in blue. References in author-date format refer to the reference list at the end of this document. In addition to thoroughly responding to each comment, we list all associated changes to the manuscript along with the corresponding line numbers (line numbers refer to the clean copy of the revised manuscript unless otherwise specified). We also use tracked changes in the revised manuscript to indicate changes made in response to Reviewer and Academic Editor comments, for ease of identification.

We hope that, based on these revisions, you will find our manuscript suitable for publication in *PLOS Biology*.

Yours sincerely,

Daniel Kronauer (on behalf of all authors)

REVIEWERS' COMMENTS:

Reviewer #1: [identifies himself as Peter Nonacs]

I very much like this paper for matching behavioral outcomes to mathematical outcomes from versions of varying complexity. Thus, when observed behavior does not match predicted behavior, the models can have biologically reasonable features added them to see what might account for the deviation. This is a very powerful integration of test and theory.

We are grateful to the Reviewer for his enthusiasm for our study.

Most of my concerns here are with how meaningful are the observed behaviors under the very simplified world of the lab experiments. A lot of this might be answered by providing a fuller account of the natural history of *O. biroi* (as I and probably most of the readers will not be familiar with this species). For example, it is described as a "raider" ant which brings to mind colonies of thousands to millions of ants. Is this true? The authors draw very small subsets of individuals from large stock colonies. How large were these stocks?

We thank the Reviewer for raising this crucial set of questions, which we address in detail below but also, as suggested, expand on in the text. We agree that a fuller account of the natural history of *O. biroi* would be helpful and would also address several of the specific comments and questions brought up here and by other Reviewers below. We have added such an account in the introduction (see details in our response to specific points below).

Questions:

(1.1) The size of the experimental colonies ranged between 8-16 workers. How does this compare to sizes in the field? How does this species start new colonies? In short, would we expect to ever see sizes this small? If not, why would we expect "typical" behavior in the lab? There is certainly a fair amount of evidence in other species that DOL is greatly affected by worker numbers.

Clonal raider ant colonies collected in the field range from a dozen to a few hundred workers (Tsuji and Yamauchi 1995; Ravary and Jaisson 2002; Trible et al. 2020), i.e. are typically somewhat larger than colonies used in our experiments, but orders of magnitude smaller than colonies of mass-raiding army ants (with 10^4 to 10^7 ants). Clonal raider ant laboratory stock colonies can grow to 10^4 workers or more, but this does not reflect the biology of the species in the field. Colony reproduction has not been observed in the field, but given that this species is queenless and asexual, it seems likely that new colonies originate when fragments of larger colonies bud off.

While the experimental colonies used here are at the lower size range or somewhat smaller than colonies in the field, previous work has established that small laboratory colonies of ca. 10 workers have high fitness and display complex collective behavior, including stable division of labor (Ulrich et al. 2018) and group raiding behavior (Chandra et al. 2020). Unlike army ants, clonal raider ants hunt in scout-initiated group raids, in which a scout recruits a modestly sized raiding party (ranging anywhere from a couple to a couple dozen workers) upon encountering prey. We chose the group sizes used here based on this previous work. We have added a section in the manuscript (L. 112) to clarify these points, as suggested by the Reviewer.

(1.2) What were the colonies fed? I'm assuming that foraging is not raiding other species in the lab. If foraging is just going a short distance to pick up food just laying there, how might this affect DOL?

The standardized feeding regime is mentioned in Materials and Methods (L. 452): *"Every 3 days, we cleaned and watered the plaster, and added one prey item (live pupae of fire ant minor workers) per live O. biroi larva at a random location within the Petri dish."* Recent work (Chandra et al. 2020) has shown that group raids (i.e., scout-initiated recruitment of workers to an undefended food source) can occur under experimental conditions very similar to the ones used here (i.e., in colonies of the same size, fed

with the same food, and foraging over similar distances of less than 10 cm). This makes it plausible that raiding, as defined in Chandra et al. (2020), also occurred in our experiments, but we could not establish this with certainty with our data collection procedure (long-term scan sampling instead of short-term continuous recording, the latter being necessary to observe raiding). Furthermore, there were small differences between the two experiments (e.g., nest box architecture, size of individual prey items). As with any experiment, the results are of course contingent on the precise experimental conditions, and it is impossible to know whether and how these small differences could have influenced foraging behavior. While the behavior observed in the lab is therefore less complex than what would happen in nature, it is likely also more complex than going a short distance to pick up food. However, these intricacies do not affect the ability of our behavioral assay to capture variation in the propensity of ants to perform tasks away from the nest, which has been shown in another study to be a good proxy for foraging activity (Ulrich et al., 2018).

(1.3) The worker vs intercaste comparison is described as just varying size. However, I assume an 'intercaste' is cross between a worker morphology and what was once in the past, a queen morphology. Given this, wouldn't one expect an intercaste to be simply less efficient at both brood and foraging? If so, their existence is better explained as group-level need to be more reproductively fecund rather than better at any task other than laying eggs. What is 'usual' frequency of intercastes in natural nests? Do you ever see any pure intercaste colonies of O. biroi? In short, I not sure how meaningful this manipulation is for explaining DOL.

The term “intercaste” really is a misnomer. The term was introduced in early papers on the species. Later, we used the term “high reproductive individual” (vs. “low reproductive individual”) (Teseo et al. 2013, 2014), but having to repeat this term in a text became too tedious. In reality, intercastes are simply larger workers, and the difference between regular workers and intercastes in *O. biroi* is much less pronounced than that between small and large workers of ants with truly polymorphic worker castes. Also, the queens of closely related species, such as *Ooceraea octoantenna*, look very different from *O. biroi* intercastes, while *O. biroi* intercastes fall within the worker size range of closely related species (Zhou et al. 2020).

Intercastes typically represent a small fraction (3.7- 6.3%) of individuals in laboratory stock colonies, (Ravary and Jaisson 2004), but that fraction can vary considerably across genotypes (Teseo et al. 2014), as well as over time within a given colony. While we never see cohorts composed exclusively of intercastes in laboratory stock colonies, we occasionally observe cohorts with very high proportions of intercastes (≥ 50%).

The Reviewer is correct that the worker vs. intercaste distinction is about more than size, and also includes variation in other morphological traits and reproductive physiology. However, unlike queens, intercastes can perform all tasks needed for colony survival and growth. For example, in our experiments, colonies composed only of intercastes reared larvae as successfully as colonies composed only of regular workers (Fig R1), indicating they are at least able to forage and nurse well enough to allow the colony to grow. Even if intercastes were not performing much of either foraging or brood care in unmanipulated colonies (which has not yet been rigorously established), the premise of the threshold model is that there exists a set of conditions (e.g., when too few regular workers are around to keep stimulus intensity below the response threshold of the intercastes) under which they would. In line with this prediction, our results suggest that intercaste behavior is more worker-like when regular workers are absent (Fig 2e).

We have added a sentence summarizing some of these points in the Introduction (L. 105).

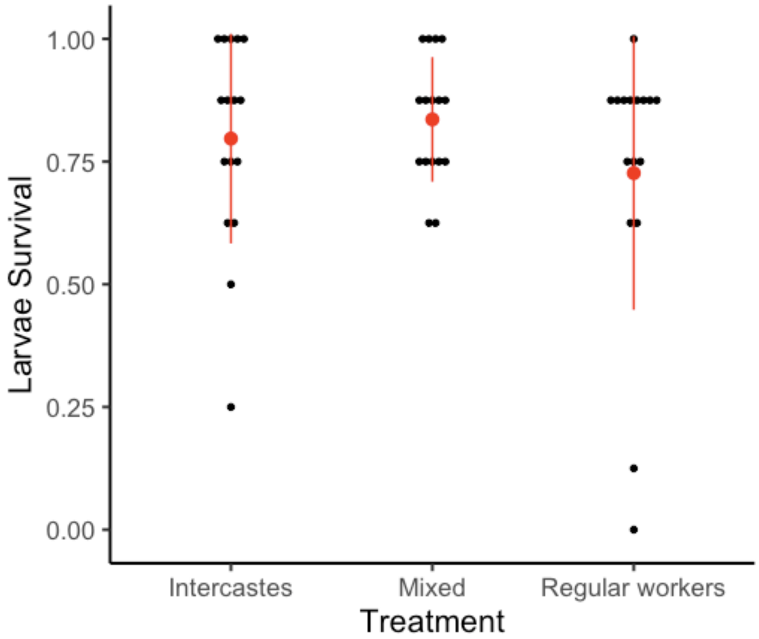

Fig R1. Larvae survival to adulthood in colonies of different morphological composition. Larvae reared by intercastes, regular workers, or a mix of intercastes and regular workers have similar survival rates ($Survival^3$ ~ Colony composition; likelihood ratio test: χ^2=1.41, p=0.49).

(1.4) How is age handled in the experiment and in the model. One of the main explanations for DOL is an age-based change in individual thresholds that goes from a bias for brood care towards doing the more dangerous foraging. In the model, it is assumed that individual thresholds are fixed over a time frame such as that of the experiment. No argument with that. However, how does model handle situations in the mixed condition? Are different ages assigned different thresholds? (In which case, it would be no surprise to produce DOL!) Overall, the experiments do seem to find patterns that age based transitions are important in creating DOL, but the authors do not seem to want to explicitly conclude that. I do realize that there are reviewers that bridle at any suggestion that age determines task, but….

We agree that age-modulated change in thresholds is an important, and already recognized, mechanism for DOL. And, consistent with this prior work, we did find patterns of age-based DOL empirically (Fig 2d). In terms of the modeling, the Reviewer is correct that we modeled the experimental setting with two different age groups by assuming that the two types differed in the mean threshold (in addition to task efficiency) (L. 351-352), and, unsurprisingly, we theoretically confirmed the emergence of DOL in this scenario (S5 and S6 Figs). Since our empirical findings on age-based DOL are consistent with the existing, well-documented findings in other social insects, and the theoretical confirmation is not surprising, we chose to only comment briefly to this effect (L. 264, *"That ants of different...age [33,34,64] differ in their task performance is consistent with observations in other social insects"*).

(1.5) Since the authors appear to know what each individual does, have they considered just analyzing the greatest outliers in each colony? Would the patterns of the 'most specialized' be more in line with predicted effects of the variables they test?

We are not entirely sure what the Reviewer means here. We interpret the first question to be asking whether we considered analyzing only those individuals with the largest (i.e., most forager-like) or smallest (i.e., most nurse-like) values of r.m.s.d. within a colony. However, we are not sure how this analysis would be useful for understanding to what extent the model recapitulates the data, particularly since we focus on the qualitative, not quantitative, recapitulation.

There may be some confusion regarding our definition of specialization: empirically, we define specialization as the *"mean correlation in individual r.m.s.d. across time"* (L. 242) and measure it as "*the Spearman correlation coefficient between individual r.m.s.d. ranks on consecutive days of the brood-care phase, averaged over days"* (L. 478-479). In other words, in our empirical analysis, we consider a colony to be more specialized if the relative ordering of r.m.s.d. values among its members is more consistent across days. Consequently, we consider specialization to be a collective, rather than an individual property (L. 240-243), though an individual that belongs to a colony with a high degree of specialization is indeed likely to be more consistent in its r.m.s.d. rank (and therefore in its relative propensity to spend time away from the nest) within the colony relative to an individual who belongs to a less specialized colony. We wonder if this is the sense in which the Reviewer considers individuals to be more "specialized"? Pre-emptively, to avoid ambiguity, we have tried to add throughout the main text the words "colony-level" or "collective" when discussing specialization. Moreover, we have introduced a new paragraph before the first set of results (L. 175-187) to further clarify the definitions.

(1.6) Finally, a small point. If we read the manuscript linearly, we encounter "r.m.s.d" before it is defined (in the Methods). It would be helpful to define it where it first appears in the text.

In the main text "r.m.s.d." is spelled out as "root-mean-square deviation" at its first occurrence at L. 220. Within Materials and Methods as well, "r.m.s.d" was spelled out as it first appeared at L. 319 of the original submission. We therefore assume that the Reviewer is referring to the mathematical definition of r.m.s.d, which we now include in the main text (L. 229), along with a more detailed explanation of its biological interpretation. We agree that this should make for a more self-consistent reading.

Respectfully submitted,

Peter Nonacs

Reviewer #2:

In this manuscript, the authors test the ability of an initially simple response threshold model to predict observed patterns of division of labour within colonies of a clonal queenless ant species. Division of labour is quantified using movement patterns, following the basic assumption that foragers travel far and wide while those engaged in nest-based tasks do not. The behaviour of colonies containing two "types" of ants is used to test the model, where colonies are composed entirely of one type, entirely of the second type, or a mixture of the two types. The nature of the "type" is varied- it can be two morphological variants, two age-cohort variants or two genetic variants. On finding that the simple model, which relies only on variation in response thresholds between types, does not provide a good fit to their findings, the authors expand the model post-hoc. Once variation between types in larval demand behaviour and in worker efficiency at particular tasks are incorporated, the fit is much better. Thus, we can conclude that types must vary not only in response threshold, but also in the latter two factors, to produce observed patterns of division of labour.

The system is indeed extraordinarily well-suited to the target question, as the authors claim, and the experiments appear well designed. My review focuses on the design and interpretation of the experiments that test the model predictions, rather than the development of the model itself (as an empiricist the latter is beyond my expertise). My criticisms are not major, but they mostly relate to the clarity of the manuscript, which in some places render the validity of the conclusions difficult to judge. Revision to improve the clarity in several places should make this task easier. Line by line comments are below.

(2.1) Title: Doesn't really tell me anything about the findings! This title is very abstract.

We agree with Reviewer that the title could be more informative and have changed it to “Response thresholds alone are insufficient to explain empirical patterns of division of labor in social insects”.

(2.2) Lines 82-85: On first reading, it wasn't clear to me why the fact that there are several axes of individual variation was a problem, given that such variation could well correlate with response thresholds. After looking into the cited references, it became clearer but I'd suggest taking more time to explain your reasoning here, given that the target journal is non-specialist.

This is a good point. We have revised our explanation as suggested (L. 83-94, new text is underlined): *“Empirically, worker behavior in social insect colonies often correlates with individual traits [16]...Such behavioral variation is often attributed to <u>the developmental or genetic modulation of</u> response thresholds. <u>However, empirical evidence suggests that</u> response thresholds are only one of several axes of possible individual variation. For example, workers can also vary in the efficiency with which they perform tasks [45–47] or in the average time spent performing a given task [48]. <u>These empirical findings suggest that previously underexplored parameters other than response thresholds may vary depending on developmental or genetic factors and may play a role in colony organization. This possibility has led</u> to recent calls for a diversity of parameters to be considered when investigating the relationship between colony composition and division of labor [16,49,50].”*

(2.3) Line 112: It would be helpful here to briefly outline a little more of the model here, in order that these predictions can be understood. While the description in the methods is clear, it isn't possible to understand this section fully without first reading that. For example, you indicate what is meant by "type" later, but it needs some explanation when first encountered. Plus, could you explain why the model predicts that mixed colonies will have more specialists? As I understand it, a specialist is an individual with a low response threshold for a particular task. In cases where the mean response threshold is higher for Type Y than Type X, across both tasks, why will there be more specialists in mixed colonies? More clarity is needed here, again, with a general audience in mind.

We appreciate and agree with the Reviewer’s point that we could better guide the reader by integrating key elements of Materials & Methods into the main text. In light of both this comment and comment (4.2) below, we have moved the model description to the beginning of Results and Discussion.

As for why the model "predicts that mixed colonies will have more specialists," we believe that there may be confusion regarding our technical definition of specialization. As discussed also in response to (1.5) above, we consider specialization to be a collective, rather than individual property, quantifying the behavioral consistency of colony members over time. Specialization is operationalized in the simulations as *"the Spearman rank correlation on consecutive windows of 200 time steps"* (L. 186, 594). DOL is also a colony-level property, characterized by both inter-individual behavioral variation (*"standard deviation of task performance frequency across individuals in a colony"*; L. 184, 588) and specialization: a colony is said to exhibit greater DOL if individuals are not only 1) more varied in their task performance (higher behavioral variation) but also 2) more consistent (higher specialization). While we had previously noted these definitions in relevant figure legends, we acknowledge that perhaps these terms were not as clear as they could have been in the original submission. We have now explicitly defined them in the Results and Discussion (L. 175-187).

The Reviewer is correct that an individual with a lower response threshold for a particular task is more likely to perform that task. Consequently, when the mean response threshold is higher for Type Y than for Type X for a task, Type X will tend to take up that task more often than Type Y, resulting in both greater colony-level behavioral variation (some individuals will be performing tasks often, others much less so) and greater colony-level specialization (individuals who are performing a particular task in a given time step are also likely to be performing that task in another time step because they are likely more sensitive to the stimuli for that task) relative to pure colonies. This is the sense in which the model predicts that a mixed colony with a wider distribution of thresholds will exhibit *"more pronounced DOL"* (L. 196). In other words, although Type Y may be more likely to remain inactive than Type X (i.e., Type Y may 'specialize' in inactivity), their behavior contributes to the measurement of colony-level DOL. To improve clarity, we have elaborated on these results in Results and Discussion (L. 189-202).

(2.4) Line 124/293-297: Why the change in colony size from 16 to 8, and corresponding increased number of replicates, for the morphological mix?

Experiments were performed separately over ca. one year. For each experiment, there was a trade-off between colony size and replicate colony number, as well as constraints on the number of slots available in the tracking system at the time each experiment was performed (experiments occasionally overlapped). The opportunity to perform the morphology experiment presented itself only once (when a stock colony produced a cohort with a unusually high rate of intercastes, which are otherwise rare (Ravary and Jaisson 2004)), over the time this entire study took place. Because such cohorts are rare, at the time, we chose to maximize the number of replicate colonies rather than colony size. This led us to allocate workers the way we did, and we realize that, in hindsight, it would have been preferable to perform the morphology experiment with 8 colonies of 16 ants instead of 16 colonies of 8 ants per treatment. Unfortunately, the opportunity to repeat that experiment did not arise.

However, given that all four experiments are analyzed independently, we believe the conclusions we are drawing are not affected by the variation in group size across experiments. We also emphasize that all group sizes used here were shown in previous work to be sufficient for stable division of labor to emerge among nestmates (Ulrich at al., 2018). We have added two sentences to Materials and Methods explaining why colony size and replicate numbers varied across experiments (L. 446).

(2.5) Line 354: Please state which response variables were transformed and which transformations were used (because it aids replication attempts based on the raw data)

The information about variable transformation was included in Materials and Methods (L. 499): *"When needed, the response variable was transformed (r.m.s.d^2 in the genotype experiment with brood of genotype A and the age experiment, r.m.s.d$^{3/5}$ in the genotype experiment with brood of genotype B; no transformation for the morphology experiment) to satisfy model assumptions."* For consistency, we have also included information about what response variables were transformed in the subsequent section (L. 511): *"Behavioral variation was square-root transformed in the genotype experiment with B larvae to satisfy model assumptions."*

(2.6) Line 340- end of statistical analysis section: Did you perform model selection, and if so, how?

Model selection was only performed to the extent that the terms of interest (one term per model in both cases) were dropped from the model to evaluate their significance. The information regarding this procedure, which was missing from the original submission, has been added at the end of the corresponding section (L. 501). *"We evaluated the significance of terms by comparing pairs of nested models using $\chi 2$ log-likelihood ratio tests following deletion of the term of interest (the interaction in the first model, and the four-level variable combining colony composition and individual attributes in the second model) using the function drop1 in R."*

(2.7) Line 349: Could you explain the logic behind including both LME models, rather than simply one? I can see why you would perform the second one, if you specifically wished to evaluate the pairwise differences between groups, and perhaps you also specifically wanted a parameter estimate for the overall effect of either Pure/Mixed or Type. But if this is the case, please make your logic clear.

The first model was used to explicitly test for an interaction between pure/mixed and type. An interaction here means that type-specific behavior depends on colony composition, which we viewed as a prerequisite to then test for pairwise differences between specific pairs among the 4 treatment groups. The second model is functionally equivalent to the first, the only difference being in the parameterization of the fixed effects, which is set up to simplify the use of the *glht* function for pairwise tests. The only difference is therefore in what specific hypotheses can be explicitly tested on each model: in the first model it is straightforward to test whether the two predictors interact (by deleting the interaction using the function *drop1*, see our reply to comment (2.6) above), while in the second model there is no explicit test for this. For completeness, we chose to report both models, rather than only one of the two. We acknowledge that the logic of the procedure was not sufficiently clear in the original submission and have striven to clarify the corresponding section of Materials and Methods in the revised submission ("Effects of individual traits on behavior" starting at L. 487). However, since we still only report and discuss the output of the second model in the main text, and if the Reviewer thinks that mentioning both models is confusing and/or redundant, we would be happy to remove the first model from the MS.

(2.8) Line 362-369: I struggled to understand both the aim and method behind this analysis. The stated aim is "to assess whether type-specific behaviour was affected by colony composition"- but is this not achieved by your LME model described above at line 349, and by the initial LME model too? A significant interaction effect in the initial LME would confirm that the effect of type on behaviour depended upon colony composition.

We thank the Reviewer for pointing this out. They are entirely correct that to assess *whether* type-specific behavior was affected by colony composition is achieved by the first LME. The goal of these analyses was to assess *how* type-specific behavior was affected by mixing, and more specifically how between-type differences in behavior were affected by mixing, i.e. whether there was behavioral contagion, divergence, or neither. This cannot be achieved simply by the first LME, because there are scenarios in which a significant statistical interaction between type and colony composition does not translate into behavioral contagion or amplification (e.g., a case where mixing simply increases the mean r.m.s.d. of each type with the same magnitude). We have changed the phrasing of that section (starting at L. 514) accordingly. It now reads: *"To assess how type-specific behavior was affected by mixing and, more specifically, whether between-type differences in behavior were affected by mixing, we compared the difference in mean behavior (type-specific mean r.m.s.d. in each colony) between types across pure colonies to the difference in mean behavior between the same types within mixed colonies, […]."*

(2.9) And when you say "Yp-Xp vs Ym-Xm", how did you match up the colonies? For each experiment, you had 8 colonies per replicate, so presumably there are 16 data points being compared in the t-tests, e.g. Yp1- Xp1, Yp2- Xp2…. Yp8- Xp8. It's not clear how the colonies were allocated into these pairs. Apologies if I have misunderstood, but this comparison is really not at all clear to me.

That is an excellent question and we appreciate the Reviewer thinking about our analyses in such detail. In mixed colonies, for Ym-Xm, the samples are "naturally" paired (the difference is calculated between, e.g., old and young workers from the same colony). For pure colonies on the other hand, there is no natural pairing between samples (because ants of different ages are by definition hosted in different colonies), so the numbers 1 to 8 in Yp1- Xp1, Yp2- Xp2…. Yp8- Xp8, are simply the unique colony identifiers assigned to colonies randomly at the start of the experiment. We cannot think of a way this could introduce any form of bias in our results but would be happy to discuss this further with the Reviewer. We have added a sentence to make this approach clearer in the corresponding Methods section (L. 519): *"In mixed colonies, the difference in mean behavior was calculated between types of ants within a colony (e.g., old and young workers from the same colony); in pure colonies, the difference in mean behavior was calculated between arbitrary pairs of pure colonies (e.g., old workers from the pure colony #1 and young ants from pure colony #1, where 1 is a replicate number assigned randomly at the beginning of the experiment)."*

(2.10) Figure 2: It isn't immediately clear how to read the curly brackets because they don't always line up correctly with the data points (e.g. Figure 2b), nor are the x-axis always well aligned with the data points (could you perhaps group the "mixed" results further away from the "pure" ones? This would help in that respect). Generally, I don't find it helpful to have all the statistical results placed in the figure legend. It is not always clear which comparisons you are referring to when you make statements in the text (e.g. line 140-142). Could you put the relevant result in the text? The link between Figure 2, the reported results in the text, and the statistical comparisons, is not at all clear.

We acknowledge that Fig 2 was very busy and agree with the Reviewer that moving some results from the figure and legends to the main text will increase clarity.

The curly brackets did not line up with data points because they represent a difference between differences between data points, not a difference between data points (this was represented by the fact that the curly brackets lined up with the grey straight dashed brackets). However, following the Reviewer's suggestion, we have opted to move these results to the main text, and these brackets are therefore no longer shown in Fig 2. To further simplify the figure, we have additionally moved the results comparing ant types in pure vs. mixed colonies (represented by straight black brackets in the original version of Fig 2), along with the corresponding statistical results, to the main text. This means there is only one type of statistical results left in the figure itself: the results showing amplification vs. contagion vs. no effect in the figure itself (black dashed lines and black curly brackets), as well as corresponding statistical results in the figure legend. Following the Reviewer's suggestion, we have also modified the layout of Fig 2 to better separate the pure and mixed colony data along the x-axis.

Reviewer #3:

In this work, the authors combined experimental and theoretical approaches to investigate how the heterogeneity in group composition shapes division of labor. Based on a qualitative agreement between experimental data and theoretical predictions, the authors concluded that the variation in response thresholds is not sufficient to account for the observed patterns of division of labor.

(3.1) First of all, I regret that the authors did not refer to previous work that studied the role of the social context, in particular the heterogeneity of phenotypes, on the division of labor in social insects. In particular, it would have been relevant to cite and discuss some of Fewell's studies on the division of labor in associations of ant foundresses or bees. Duarte's theoretical work also contains elements relevant to those found in the present study.

We appreciate the Reviewer's attention to the contextualization of the paper, and we wholeheartedly agree that Jennifer Fewell's work is both important and extremely relevant, which is why in the original submission we cited three of her papers that we thought most closely pertained to our own study:

*one empirical study on division of labor in associations of bees (Holbrook CT, Kukuk PF, Fewell JH. Increased group size promotes task specialization in a normally solitary halictine bee. Behaviour. 2013;150: 1449–1466),

*a theoretical study on thresholds models of DOL (Jeanson R, Fewell JH, Gorelick R, Bertram SM. Emergence of increased division of labor as a function of group size. Behav Ecol Sociobiol. 2007;62: 289–298).

*Fewell's classic review on DOL in social insects (Beshers SN, Fewell JH. Models of division of labor in social insects. Annu Rev Entomol. 2001;46: 413–440), which in turn contains references to her earlier empirical studies on ant foundress associations.

We recognize that this is far from an exhaustive coverage, but we had felt this would adequately cover the directly relevant aspects of Jennifer Fewell's work, which is why we are a bit puzzled by the Reviewer's comment. It was certainly not our intention to downplay the importance of this work. Consequently, in response to the Reviewer's comment, we now cite additional work by Jennifer Fewell on emergent DOL in forced associations of ant queens (L. 81) (Fewell and Page 1999; Jeanson and Fewell 2008), on genotypic effects on foraging propensity in honeybees (L. 84) (Fewell and Page 1993) and on the effect of social interactions on foraging in honeybees (L. 387) (Fewell and Bertram 1999).

Regarding Ana Duarte's work, we are less certain of its immediate relevance to our study. Her theoretical work on DOL focuses on the evolutionary processes generating variation in thresholds and on the adaptive value of different threshold distributions (Duarte et al. 2011, 2012a, b). While we believe that this work falls outside the scope of our study, which focuses (both theoretically and empirically) on the short-term, proximate drivers of DOL, we think that the review by Duarte et al. (Duarte et al. 2011) in fact provides a helpful perspective on how our work could inform future studies on the evolutionary trajectories of DOL, and we now include it in the Introduction (L. 64) and the Conclusions (L. 370).

(3.2) I also regret that the authors did not discuss their results more comprehensively. I would have appreciated more in-depth comments on the mechanisms underlying the observed patterns. In particular, the authors only briefly describe the results of the exploration of their model but without providing any substantial interpretation. As things stand, the argument of the recapitulation found in the mixed groups (Fig. 3) was not enough to convince me that differences in task performance efficiency or changes in demand (lines 182-183) were at work in ant colonies. This certainly opens up some interesting avenues for future research but I believe that additional data should be collected (or presented) to give more credit to this hypothesis and thus grant publication in PloS Biology.

First, we agree that the results could be discussed more comprehensively. To that end, we have expanded the discussion by elaborating on our theoretical predictions for colony compositions that were not used in experiments (e.g., different ratios of different ant types; S7 Fig, L. 389-398) and including mentions of other theoretical models and empirically documented processes that were not considered in our study (L. 387-389).

Second, we of course do not claim that our theoretical results alone can definitively demonstrate that differences in task performance efficiency or changes in demand are at work. All a theoretical model can do is propose candidate mechanisms and make predictions that, as the Reviewer suggests, then get further tested empirically; we now note this explicitly in L. 377. Unfortunately, in social insects, explicitly linking model parameters to empirical measurements is extremely challenging. For instance, as of right now, there is no simple way to measure variation in individual nursing efficiency or in larval demand; doing so would require designing entirely new behavioral assays. In fact, even response thresholds—a well-accepted mechanism for DOL that has been around for decades—have very rarely been measured empirically (Detrain and Pasteels 1991; Weidenmuller 2004); and even when they have been measured, the link between stimulus, task, and DOL in a colony context is not always clear (Merling et al. 2020; Pankiw and Page 2000). To our knowledge, there is therefore no more empirical evidence for individual variation in response threshold than there is for variation in efficiency, for example. We now make these challenges more explicit in the main text at L. 380.

That such empirical work should be done in the future is indisputable and, in that respect, we agree with the Reviewer and highlight this in our Conclusions (L. 380-383). However, we disagree that an inability to presently carry out such empirical work undermines the main findings of our study, which are that i) behavioral response thresholds alone cannot explain our results, and ii) adding variation in parameters *that are known* to vary in nature is sufficient to explain the results. This does not mean that variation in these parameters is the answer (or the whole answer). But it does move the discussion forward from the standard explanation of behavioral organization based on variation in thresholds alone to one that considers a more complex set of factors (including e.g., task performance efficiency and task demand). This, in turn, also expands the explanatory power of the theoretical framework. We believe that the theory-experiment dialog in our study provides ample basis for future empirical and theoretical research on DOL across systems.

(3.3) Overall, although I appreciate that the division of labor is a common property of sociality, I doubt that this work will be of interest for a large audience. I would recommend the authors to submit this work to a more specialized journal.

We respectfully disagree and, importantly, so do the other Reviewers. Reviewer 4 highlights that the central question of our study—how group composition affects collective behavior—is "[an] important [topic] not just to behavioral biologists, but also to social scientists and employers more broadly." Reviewer 1 comments that our approach—"matching behavioral outcomes to mathematical outcomes from versions of varying complexity"—offers "a very powerful integration of test and theory." Moreover, the parameters and their interactions studied here—task efficiency, task demand, and response thresholds—are central to our understanding of not only social insects, but complex biological and artificial systems in general. These include anything from neural circuits and microbial communities to human social networks and robot swarms. As Reviewer 4 notes, "[t]he incorporated concepts of task efficiency and stimulus intensity are useful and I hope this model will be applied in other situations."

To emphasize the broad relevance of this work, we have added a paragraph in Results and Discussion elaborating on the general implications for complex systems science (L. 400-411).

(3.4) As minor comments, the authors should mention what were the initial conditions of each simulation in terms of stimulus levels.

The lack of initial conditions was an accidental omission on our part and we are grateful to the Reviewer for spotting that. Both stimuli were initialized to zero at the start of each simulation run. We have updated the model description accordingly (L. 162).

(3.4, continued) Also, it would also have been useful to provide information on the evolution of the stimulus level for each task when manipulating demand (Fig 3), as the authors wrote "when the demand was so high, the least effective type could not keep up with the demand on its own" (lines 188-189).

Great question. We have now introduced a new SI figure, S4 Fig (cited in L. 321 and included below) that tracks stimulus dynamics. When the demand[1] was higher (δ = 1.3, as in Fig 3a), the more efficient, Type X, individuals could still keep up with the stimuli on their own (S4a Fig; stimulus levels stabilized to an oscillatory pattern around a finite level) but the less efficient, Type Y, individuals could not (S4b; stimulus levels kept growing). However, mixed colonies of Type X and Type Y could keep up with the demand (S4c Fig), suggesting that the more efficient Type X could compensate for the inefficiency of Type Y in mixed colonies. On the other hand, when the demand was lower (δ = 0.6, as in Fig 3b), both Type X and Type Y individuals could keep up with the demand on their own (S4d,e Fig), as could mixed colonies (S4f Fig).

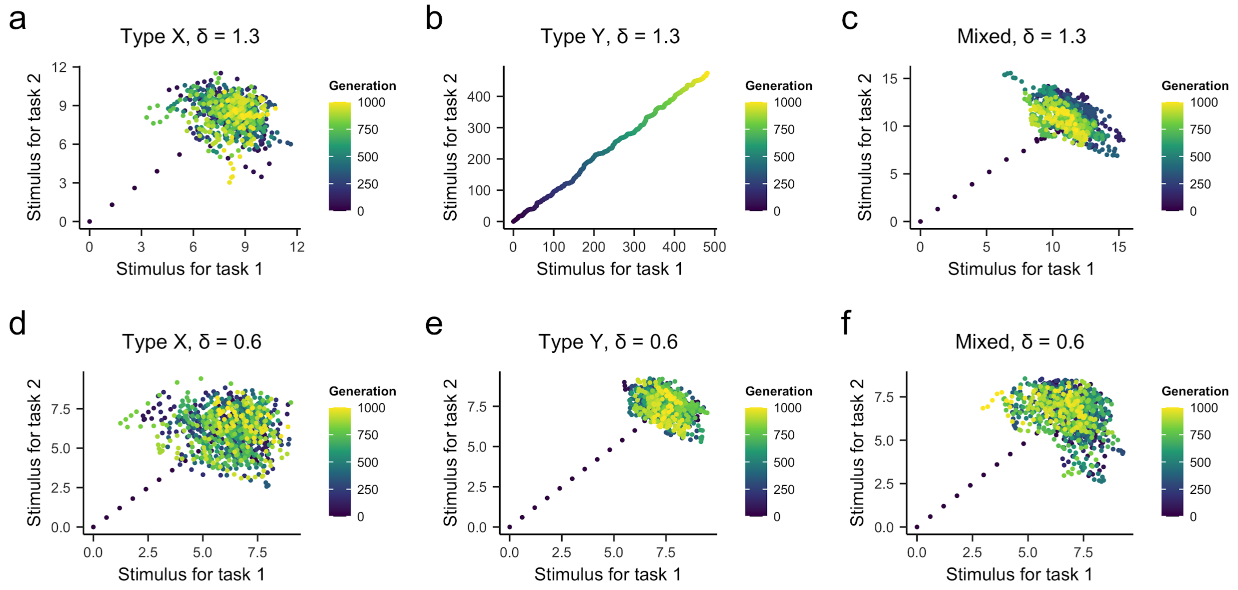

S4 Fig. Dynamics of stimulus levels in pure and mixed colonies. Each point shows the simulated stimulus level for the two tasks (task 1 on the horizontal axes, task 2 on the vertical axes) in the generation indicated by its color. Each of panels **a, b, d,** and **e** shows a pure colony of the type indicated; each of panels **c** and **f** shows a mixed colony of Types X and Y. Panels **a-c** (δ = 1.3) correspond to Fig 3a and **d-f** (δ = 0.6) to Fig 3b. **a-c**: When the demand is higher (δ = 1.3), the more efficient type (Type X) can keep up with the demand on its own (**a**) but the less efficient type (Type Y) cannot, as demonstrated by the continual growth of the stimuli (**b**); however, mixed colonies can keep up with the higher level of demand (**c**). **d-f**: When the demand is lower (δ = 0.6), the stimulus levels grow quickly at first but then stabilizes to an oscillatory pattern around a point, demonstrating that both pure and mixed colonies can keep up with the demand. Each simulation ran for 1000 time steps; all other parameters are identical to those in the corresponding panels in Fig 3.

(3.4, continued) With a high level of demand for one task, were simulated workers able to cope with the two tasks?

While in the simulations we assumed that the two tasks had the same demand, our analytical calculations in the SI allow for the possibility that the demand differs between tasks. They reveal that having one high-demand task and one low-demand task would be intermediate between the more extreme scenarios (i.e., having two high-demand or two low-demand tasks), both in terms of task performance and in terms of the colonies' ability to keep up with the demand, which is why in the simulations we only considered the extreme scenarios. We now add a sentence on these analytical insights in the Results and Discussion (L. 322-325).

---

[1] In all simulations in the paper we have assumed that both tasks have the same demand ($\delta_1 = \delta_2 = \delta$) but see our answer in response to your last question for predictions when this assumption is relaxed.

Reviewer #4:

The authors use a clonal ant to test long-standing ideas about the effect of group composition on division labor. They found that the simple, often touted models based only on behavioral thresholds are insufficient to capture the variability of this the observed behavior. When they then added variation in task efficiency and task demand between types they could recapitulate the observed behaviors.

The story is well-conceived, the research elegantly done and the paper well-written.

How group composition impacts division of labor and group-level efficiency are important topics not just to behavioral biologists, but also to social scientists and employers more broadly. The use of this species to answer this question is novel and solves problems in experimental design that could not be addressed in other ways. The incorporated concepts of task efficiency and stimulus intensity are useful and I hope this model will be applied in other situations.

We thank the Reviewer for these encouraging comments!

Major (not necessarily major, but should be addressed):

(4.1) *Why is the morphology experiment only done with 8 individuals and 16 times (while the other experiments had 16 individuals, 8 times)?

This is an important point, which was also raised by Reviewer 2 (comment (2.4)).

The four experiments presented in this study were performed separately over ca. one year. For each experiment, we faced a trade-off between colony size and replicate colony number, as well as constraints on the number of slots available in the tracking system at the time each experiment was performed (different experiments occasionally overlapped). The opportunity to perform the morphology experiment presented itself only once (when a stock colony produced a cohort with an unusually high rate of intercastes, which are otherwise rare (Ravary and Jaisson 2004)), over the time this entire study took place. At that time, we decided to maximize the number of replicate colonies rather than colony size. This led us to allocate workers the way we did, and we realize that, in hindsight, it would have been preferable to perform the morphology experiment with 8 colonies of 16 ants instead of 16 colonies of 8 ants per treatment. Unfortunately, the opportunity to repeat that experiment did not arise.
However, given that all experiments are analyzed independently, we believe the conclusions we are drawing are not affected by the variation in group size across experiments. We also emphasize that all group sizes used here were shown in previous work to be sufficient for stable division of labor to emerge among nestmates (Ulrich at al., 2018). We have added two sentences to Materials and Methods explaining why colony size and replicate numbers varied across experiments (L. 446).

(4.2) *I would have liked more of the model (including equations) in the results section. Understanding how it is built is necessary to appreciate and understand your findings! I would also like more discussion on the implications of the model and how these contagions/amplification relate to existing literature.

In light of this comment and a similar one from Reviewer 2, we have moved the description of the theoretical model to the start of Results and Discussion (L. 120-163).

In light of the second part of this comment as well as comment (3.3) from Reviewer 3, we have added a paragraph at the end of Results and Discussion highlighting the implications of our model for the theoretical literature on collective behavior in complex systems (L. 400-411).

Minor:
(4.3) *I felt that there needed to be a bit more of an opening to the results section. For example, in the first sentence of the result, the authors refer to "the model" but it is not clear which model is being referred to.

We appreciate and agree with this comment. We hope the addition of the model description at the start of Results and Discussion addresses the Reviewer's concern.

(4.4) *The phrase "between-type differences" is somewhat confusing. In places it felt like a specific term you were using that needed definition, when eventually I realized it is just a way to express consistent variation between types for a given parameter. It is also only used in the main text, but not in figures and supplement. In many cases one could cut this phrase and the sentences are easier to understand.

The Reviewer's interpretation of the phrase "between-type differences" is correct. Our intent behind this phrase was to distinguish between within-type and between-type variation in a given parameter. This distinction is often important and necessary when discussing response thresholds because they can vary both within a type (e.g., individual thresholds are drawn from distributions) and between types (e.g., the means of the distributions can differ depending on the theoretical scenario of interest). However, we agree with the Reviewer that the phrase is often unnecessary when discussing the other parameters, which are assumed to be uniform within a type. To improve clarity, we have added a parenthetical explanation following the first instance of this phrase (L. 313) and removed the phrase throughout the main text where we could do without it.

(4.5) *I would like more discussion. How does this model relate to the many other models that have been developed for division of labor? What can be deduced about forms of behavioral variation and what can be predicted? The authors are frank in that their results vary in all possible directions. What can we take away from this? Would results be different had they analyzed still other genotypes or ages?

Following the Reviewer's suggestion, we have expanded the discussion to include sections on the main contributions of our study (L. 358; see also comment (4.7) below), as well as mentions of other theoretical models and empirically documented processes that were not considered here (L. 387). We also discuss our theoretical predictions for colony compositions that were not used in experiments (S7 Fig) in more depth (L. 391) and provide a broader perspective on how our study connects to other fields of research (L. 400).

(4.6) *Age. In the lifespan of these ants, where are the ages used here (are these very old ants, very young, young-middle and old-middle, etc)?

The average lifespan of clonal raider ants has not been rigorously measured, but anecdotal evidence indicates that these ants can live a year or more (at least 10% of ants collected in the field are still alive 14 months later). In other words, our 1-month old ants are young but "self-sufficient," while 3-month old ants can be considered middle aged but not yet "on the decline." We have added this information both in Materials and Methods (L. 435) and in the main text (L. 219).

(4.7) *The authors should explain more clearly why this work could not be done with "normal" social insects.

We have clarified this point by adding a sentence in the introduction (L. 58): *"a typical social insect colony consists of one or more queens, dozens to thousands of workers of different (and often unknown) age, genotype, and morphology, and various brood development stages"*, as well as a paragraph in the discussion (L. 358): *"In most social insect colonies, all factors studied here (worker genotype, age, morphology, larval genotype) influence behavior simultaneously and in largely intractable ways. However, the unique biology of O. biroi allows us to break this complexity down experimentally to study each effect independently, thereby providing insight into the basic organizing principles of behavior in social groups. Our finding that the magnitude and direction of effects on DOL depend on the specific factor being manipulated underscores the importance of considering and controlling the various sources of heterogeneity that naturally act in social groups in order to study the different (and possibly opposing) effects that they have on collective organization."*

(4.8) *Can you look at your behavioral data included here and determine task efficiency or behavioral threshold per individual? (more of a curiosity than a request for a change in the manuscript)

Explicitly linking parameters in the threshold model to empirical measurements is extremely challenging and, as far as we know, has rarely been attempted. In the model, an individual's response threshold corresponds to the value of a stimulus at which this individual starts performing the corresponding task. Efficiency, in turn, measures by how much an individual decreases stimulus intensity by performing the corresponding task. Measuring individual response thresholds or task performance efficiency therefore requires the ability to measure (or better, control) stimulus intensity. In our system, the relevant stimulus (larval stimulation, most likely via pheromones) has not yet been characterized and could therefore not be measured. While this could in principle be done in the future, it would require designing new, dedicated behavioral assays. We now make these limitations more explicit in the manuscript (L. 380).

Resource Availability:
(4.9) *The agent-based models and stats are done in R but so far as I could tell no code was available.

This was an accidental omission on our part. All simulation code is available at https://github.com/marikawakatsu/mixing-model. Additionally, software for image analysis is available at https://doi.org/10.5281/zenodo.1211644. Behavioral tracking data (raw position data and summary statistics for each individual, colony, and subcolony), as well as R scripts for statistical analyses are available in the Dryad repository https://datadryad.org/stash/share/sCrq8wEs2df7I_KXJ8m_WxlC4nZbPO70Pb_yj_NbxFs, which will be made public upon publication. We have updated the Data Availability statement accordingly.

COMMENTS FROM THE ACADEMIC EDITOR:

I'd like the following comments to be addressed:

(AE.1) Efficiency: I could not find a clear description on how efficiency is defined or measured, and this seems really crucial.

That is, indeed, an unfortunate omission, which we have now corrected. In the model, task performance efficiency is defined as the extent to which an individual decreases stimulus intensity by performing the corresponding task. Both efficiency and threshold are assumed to be fixed over the simulation run, which corresponds to the duration of the experiment. This is now made clear in the more detailed description of the model included in the Results and Discussion section (L. 120-163). In our experiments, task efficiency would be, for example, the extent to which an individual worker decreases larval hunger levels by foraging for a given amount of time. Measuring this efficiency remains, currently, impossible because the relevant stimulus (most likely one or more pheromones that signal larval hunger) has not yet been characterized and could itself, therefore, not be measured (see also our replies to comments (3.2) and (4.8) above).

(AE.1 continued) Moreover, the authors do not consider experience as a determining factor in efficiency, which seems a significant oversight, especially since experience has been shown to generate division of labour in the same species by other authors: https://urldefense.proofpoint.com/v2/url?u=https-3A__www.sciencedirect.com_science_article_pii_S0960982207016168&d=DwIGaQ&c=JeTkUgVztGMmhKYjxsy2rfoWYibK1YmxXez1G3oNStg&r=IU1ddn38VMIsDsBk3K3mnDnZlYvIIMsyqkSrjC5oO-4&m=De_el44_vB3DPokhmuunC79XIx8HhtMROX1cdOOAVWc&s=k-LYxEvVBpl42f2P0HTXJSaUjR0Dc3tDdDfE1WN1R0w&e=

The study brought up by the AE deals with the effects of experience on DOL in the clonal raider ant. It is now cited at L. 84 in the Introduction and L. 349 as part of our expanded discussion. That study shows that when individual ants are experimentally made to discover prey at each of their foraging attempts, they then show a higher propensity for food exploration (i.e., nest exits) than unsuccessful foragers. While this finding supports the idea that experience affects individual response thresholds and can therefore generate DOL independently from other factors in the clonal raider ant, it does not deal with the effect of experience on task performance efficiency (which in this case would be, e.g., the number of prey items brought back to the nest when foraging). Therefore, it is still unclear whether experience affects task efficiency in the clonal raider ant. More generally, the empirical evidence for effects of experience on task efficiency in social insects is equivocal (O'Donnell and Jeanne 1992; Tripet and Nonacs 2004; Dornhaus 2008).

(AE.2) Contagion: I was not sure I understood their notion of behavioural contagion – this should benefit from a clearer definition.

Behavioral convergence / contagion refers to a scenario in which two types of ants behave more similarly in mixed colonies than in separation (i.e., across pure colonies); this is in contrast to behavioral divergence, which refers to the ant types behaving more differently from one another in mixed colonies than in separation. The mathematical definition of behavioral contagion is included in Materials and Methods (L. 531): *"Behavioral contagion: individuals of different types are behaviorally more similar on average to each other when mixed, so that $Y_p - X_p > Y_m - X_m$".* In addition to clarifying our definition in the main text (L. 248-250), we have also streamlined and clarified the legend of Fig 2 to make it easier to visually identify the scenarios corresponding to behavioral contagion, divergence, or neither, as well as the statistical comparisons that were performed in each experiment to establish which scenario occurred.

(AE.3) Scholarship: In addition to the above study on clonal raider ants, I agree with one of the referees that Jennifer Fewell's work on threshold models must be cited.

We agree with the Academic Editor and Reviewer 3 that Jennifer Fewell's work on threshold models is foundational to the literature on division of labor and provides important background for our work, which is

why we had, in the original submission, cited three of her papers that we considered to be most pertinent to this study:

*Beshers SN, Fewell JH. Models of division of labor in social insects. Annu Rev Entomol. 2001;46: 413–440 (a review by Fewell on models of DOL, which cites a number of Fewell's work on threshold models);

*Holbrook CT, Kukuk PF, Fewell JH. Increased group size promotes task specialization in a normally solitary halictine bee. Behaviour. 2013;150: 1449–1466 (an empirical study on DOL in associations of bees);

*Jeanson R, Fewell JH, Gorelick R, Bertram SM. Emergence of increased division of labor as a function of group size. Behav Ecol Sociobiol. 2007;62: 289–298 (a theoretical study on the impact of group size on DOL).

Thus, we are a bit puzzled by Reviewer 3's suggestion that we have ignored Fewell's work. Nevertheless, in addition to these three papers originally cited, and as noted in our reply to comment (3.1), we have now also added references to additional work by Fewell on emergent DOL in forced associations of ant queens (L. 81), on genotypic effects on foraging propensity in honeybees (L. 84) and on the effect of social interactions on foraging in honeybees (L. 348).

(AE.3, continued) The earliest version of the sensory threshold idea and division of labour dates to Francois Huber (1814): Nouvelles Observations sur les Abeilles (Second Edition). There is an English translation by Dadant available on the internet somewhere.

We thank the Academic Editor for pointing us to the pioneering work of François Huber, which we were not aware of. We have read the suggested book and now cite it in the Introduction (L. 71). In particular, we note the following section of his book, on the notion of variation in thresholds as they relate to DOL in the context of honeybees fanning their wings to circulate air in the hive (pp. 391-392, our English translation in footnote[2]): "Les insectes de même espèce, quoique excités pas une même cause, n'éprouvent pas si également son influence que l'on ne voie souvent quelque différence dans les résultats des expériences dont ils sont l'objet. Les uns en sont affectés plus promptement que les autres; telle circonstance, telle occupation les rend momentanément plus ou moins sensibles, et ce n'est quelquefois que lorsque la cause est à un degré extrême qu'elle agit sur eux avec toute son énergie. Ils se pourroit donc, que dès qu'une certain nombre de ventilatrices sont parvenues à rendre l'air d'une pureté suffisante; les autres, n'éprouvant plus au même point la sensation qui les porteroit à mettre leurs ailes en mouvement, s'exemptent de cette fonction pour se livrer à des occupations plus pressantes. Si le nombre des abeilles ventilantes diminuoit momentanément, les premières ouvrières s'apercevroient de l'altération de l'air se mettroient en devoir de s'éventer, et leur nombre s'accroîtroit jusqu'à ce que leurs efforts réunis devinssent capables de rendre à ce fluide le degré de pureté essentiel à la respiration de tant de milliers d'individus."

[2] "Insects of the same species, although excited by the same cause, do not feel its influence so equally that one does not see differences in the results of experiments they are subjected to. Some individuals are affected more rapidly than others; a given circumstance or occupation makes them momentarily more or less sensitive, and it is sometimes only when the cause is at an extreme degree that it acts on them with all its energy. It could therefore be that as soon as a certain number of fanning bees have succeeded in making the air sufficiently pure, the others, no longer feeling the sensation that leads them to set their wings in motion, forego this function in order to engage in more pressing occupations. If the number of fanning bees were to decrease momentarily, the former workers would notice the alteration of the air, would start to fan themselves, and their numbers would increase until their combined efforts restored to this fluid the degree of purity essential to the respiration of so many thousands of individuals."

References

Beshers SN, Fewell JH (2001) Models of division of labor in social insects. Annu Rev Entomol 46:413–440

Chandra V, Gal A, Kronauer DJC (2020) Colony expansions underlie the evolution of army ant mass raiding. bioRxiv. Doi: 10.1101/2020.08.20.259614

Detrain C, Pasteels JM (1991) Caste differences in behavioral thresholds as a basis for polyethism during food recruitment in the ant, *Pheidole pallidula* (Nyl.) (Hymenoptera: Myrmicinae). J Insect Behav 4:157–176

Dornhaus A (2008) Specialization does not predict individual efficiency in an ant. PLoS Biol 6:e285

Duarte A, Pen I, Keller L, Weissing FJ (2012a) Evolution of self-organized division of labor in a response threshold model. Behav Ecol Sociobiol 66:947–957

Duarte A, Scholtens E, Weissing FJ (2012b) Implications of behavioral architecture for the evolution of self-organized division of labor. PLoS Comput Biol 8:e1002430

Duarte A, Weissing FJ, Pen I, Keller L (2011) An evolutionary perspective on self-organized division of labor in social insects. Annu Rev of Ecol Evol. Syst, Vol 42 42:91–110

Fewell JH, Bertram SM. Division of labor in a dynamic environment: Response by honeybees (*Apis mellifera*) to graded changes in colony pollen stores. Behav Ecol Sociobiol. 1999;46: 171–179.

Jeanson R, Fewell JH, Gorelick R, Bertram SM (2007) Emergence of increased division of labor as a function of group size. Behav Ecol Sociobiol 62:289–298

Merling M, Eisenmann S, Bloch G (2020) Body size but not age influences phototaxis in bumble bee (*Bombus terrestris*, L.) workers. Apidologie 51:763-776

O'Donnell S, Jeanne RL (1992) Forager success increases with experience in *Polybia occidentalis* (Hymenoptera: Vespidae). Insectes Soc 39:451–454

Pankiw T, Page RE Jr (2000) Response thresholds to sucrose predict foraging division of labor in honeybees. Behav Ecol Sociobiol 47:265–267

Ravary F, Jaisson P (2002) The reproductive cycle of thelytokous colonies of *Cerapachys biroi* Forel (Formicidae, Cerapachyinae). Insectes Soc 49:114–119

Ravary F, Jaisson P (2004) Absence of individual sterility in thelytokous colonies of the ant *Cerapachys biroi* Forel (Formicidae, Cerapachyinae). Insectes Soc 51:67–73

Tate Holbrook C, Kukuk PF, Fewell JH (2013) Increased group size promotes task specialization in a normally solitary halictine bee. Behaviour 150:1449–1466

Teseo S, Chaline N, Jaisson P, Kronauer DJC (2014) Epistasis between adults and larvae underlies caste fate and fitness in a clonal ant. Nat Commun 5: 3363

Teseo S, Kronauer DJC, Jaisson P, Chaline N (2013) Enforcement of reproductive synchrony via policing in a clonal ant. Curr Biol 23:328–332

Trible W, McKenzie SK, Kronauer DJC (2020) Globally invasive populations of the clonal raider ant are derived from Bangladesh. Biol Lett 16:20200105

Tripet F, Nonacs P (2004) Foraging for work and age-based polyethism: The roles of age and previous experience on task choice in ants. Ethology 110:863–877

Tsuji K, Yamauchi K (1995) Production of females by parthenogenesis in the ant, *Cerapachys Biroi*. Insectes Soc 42:333–336

Ulrich Y, Saragosti J, Tokita CK, et al (2018) Fitness benefits and emergent division of labour at the onset of group living. Nature 560:635–638

Weidenmuller A (2004) The control of nest climate in bumblebee (*Bombus terrestris*) colonies: interindividual variability and self reinforcement in fanning response. Behav Ecol 15:120–128

Zhou S, Chen D, Chen Z (2020) Discovery of novel Ooceraea (Hymenoptera: Formicidae: Dorylinae) species with 8-segmented antennae from China. Sociobiology 67:139–143
