# Supplementary material for: Response thresholds alone cannot explain empirical patterns of division of labor in social insects
Source: PLoS Biol. 2021 Jun 17;19(6):e3001269. doi: 10.1371/journal.pbio.3001269 (PMC8211278; doi:10.1371/journal.pbio.3001269)
Supplement: Attachment — Submitted filename: Ulrich_Kawakatsu_etal_ResponseToReviewers.pdf [file pbio.3001269.s012.pdf]

Response to Reviewers

The three Reviewers were satisfied with our changes.

The Academic Editor's comment is reprinted below in plain text, followed by our response in blue. We list all associated changes to the manuscript along with the corresponding line numbers (line numbers refer to the tracked changes version of the revised manuscript). Our responses to editorial requests are also highlighted as tracked changes in that version of the manuscript.

COMMENT FROM THE ACADEMIC EDITOR (lightly edited):

I am happy with the revisions the authors have made in response to the referees. I am still not fully convinced by their definition or usage of the term "behavioural contagion" - I'd like to see a bit more on how the authors envisage the spread of information between individuals.

Do the authors have some sort of social learning in mind (cf Alem et al (2016) Associative Mechanisms Allow for Social Learning and Cultural Transmission of String Pulling in an Insect. PLoS Biology 14(10): e1002564. doi:10.1371/journal.pbio.1002564)...

...or the kind of information transfer that happens in animal swarms (Revealing the hidden networks of interaction in mobile animal groups allows prediction of complex behavioral contagion. By: Rosenthal, Sara Brin; Twomey, Colin R.; Hartnett, Andrew T.; et al. PNAS Volume: 112 Issue: 15Pages: 4690-4695 Published: APR 14 2015)?

In brief, what are some plausible mechanisms of information spread? This will just take 15 minutes to address, but I'd like to see this discussed.

As we understand the Academic Editor's comment, there seems to be a semantic confusion regarding our definition of the term "behavioral contagion". In our definition, behavioral contagion occurs when "genotypes [behave] more similarly in mixed colonies than in separation (i.e., across pure colonies)" (L. 274 in the main text), which corresponds mathematically to cases in which "individuals of different types are behaviorally more similar on average to each other when mixed, so that $Y_p - X_p > Y_m - X_m$," where X_k and Y_k are the mean behavior of ants of type X and Y, respectively, in pure ($k = p$) or mixed ($k = m$) colonies (L. 558 Material & Methods). **This definition makes no assumptions about the mechanism leading to behavioral contagion** and, importantly, does not require direct interactions between ants of different types. Akin to disease contagion, which does not necessarily always require direct contact, behavioral contagion can occur not only via direct interaction between individuals (as in the examples referenced by the AE), but also via indirect interactions through a common environment (in the case of the threshold model, a globally available stimulus such as a larval pheromone). **This is, in fact, a main contribution of the threshold model framework we used here:** the model was able to recapitulate the full range of experimentally observed phenomena (behavioral contagion, amplification, or lack of effect) only using simple behavioral rules and without invoking social interactions. Importantly, this means that complex phenomena like behavioral contagion can emerge without complex information transfer or social learning. We now clarify this point in the Conclusions (L. 395) and cite the two references mentioned by the Academic Editor (underlined below): "Importantly, the expanded threshold model could recapitulate these patterns using only simple individual behavioral rules, and without invoking social interactions. For example, behavioral convergence—a phenomenon that intuitively appears to rely on direct social interactions—could emerge without invoking complex social processes, such as social learning (Alem et al. 2016; van de Waal, Borgeaud, and Whiten 2013) or direct information transfer between group members (Rosenthal et al. 2015; Berdahl et al. 2013)."

However, we recognize that no matter how hard we try to clarify the definition that we employ, the term "contagion" itself could be interpreted by some readers to *imply* that direct social interactions are

involved. To pre-empt such potential confusion, we have replaced all instances of "behavioral contagion" with the much more mechanism-agnostic "behavioral convergence" throughout the manuscript. For symmetry, we have also replaced all instances of "behavioral amplification" with "behavioral divergence". Together with the clarifying sentence incorporated in the Conclusions, this should eliminate the potential for confusion.

References

Alem, Sylvain, Clint J. Perry, Xingfu Zhu, Olli J. Loukola, Thomas Ingraham, Eirik Søvik, and Lars Chittka. 2016. "Associative Mechanisms Allow for Social Learning and Cultural Transmission of String Pulling in an Insect." *PLOS Biology* 14 (10): e1002564.

Berdahl, Andrew, Colin J. Torney, Christos C. Ioannou, Jolyon J. Faria, and Iain D. Couzin. 2013. "Emergent Sensing of Complex Environments by Mobile Animal Groups." *Science* 339 (6119): 574–76.

Rosenthal, Sara Brin, Colin R. Twomey, Andrew T. Hartnett, Hai Shan Wu, and Iain D. Couzin. 2015. "Revealing the Hidden Networks of Interaction in Mobile Animal Groups Allows Prediction of Complex Behavioral Contagion." *Proceedings of the National Academy of Sciences of the United States of America* 112 (15): 4690–95.

Waal, Erica van de, Christèle Borgeaud, and Andrew Whiten. 2013. "Potent Social Learning and Conformity Shape a Wild Primate's Foraging Decisions." *Science* 340 (6131): 483–85.
